# Microenvironment, systemic inflammatory response and tumor markers considering consensus molecular subtypes of colorectal cancer

**DOI:** 10.3389/pore.2024.1611574

**Published:** 2024-04-05

**Authors:** Anna Jakab, Árpád V. Patai, Mónika Darvas, Karolina Tormássi-Bély, Tamás Micsik

**Affiliations:** ^1^ Department of Pathology and Experimental Cancer Research, Semmelweis University, Budapest, Hungary; ^2^ Interdisciplinary Gastroenterology Working Group, Semmelweis University, Budapest, Hungary; ^3^ Department of Surgery, Transplantation and Gastroenterology, Semmelweis University, Budapest, Hungary; ^4^ Saint George University Teaching Hospital of Fejér County, Székesfehérvár, Hungary

**Keywords:** colorectal cancer, consensus molecular subtypes, tumor microenvironment (TME), tumor-stroma ratio, systemic inflammation

## Abstract

**Introduction:** Colorectal carcinomas (CRC) are one of the most frequent malignancies worldwide. Based on gene expression profile analysis, CRCs can be classified into four distinct subtypes also known as the consensus molecular subtypes (CMS), which predict biological behaviour. Besides CMS, several other aspects of tumor microenvironment (TME) and systemic inflammatory response (SIR) influence the outcome of CRC patients. TME and inflammation have important role in the immune (CMS1) and mesenchymal (CMS4) subtypes, however, the relationship between these and systemic inflammation has not been assessed yet. Our objective was to evaluate the connection between CMS, TME and SIR, and to analyze the correlation between these markers and routinely used tumor markers, such as CEA (Carcinoembryonic Antigen) and CA19-9 (Carbohydrate Antigen 19-9).

**Methods:** FFPE (Formalin Fixed Paraffin Embedded) samples of 185 CRC patients were collected. TME was described using tumor-stroma ratio (TSR), Klintrup-Makinen (KM) grade, and Glasgow Microenvironment Score (GMS). CMS classification was performed on tissue microarray using MLH1, PMS2, MSH2 and MSH6, and pan-cytokeratin, CDX2, FRMD6, HTR2B and ZEB1 immunohistochemical stains. Pre-operative tumor marker levels and inflammatory markers [C-reactive protein - CRP, albumin, absolute neutrophil count (ANC), absolute lymphocyte count (ALC), absolute platelet count (APC)] and patient history were retrieved using MedSolution database.

**Results:** Amongst TME-markers, TSR correlated most consistently with adverse clinicopathological features (*p* < 0.001) and overall survival (*p* < 0.001). Elevated CRP and modified Glasgow Prognostic Score (mGPS) were associated with worse outcome and aggressive phenotype, similarly to tumor markers CEA and CA19-9. Stroma–Tumor Marker score (STM score), a new combined score of CA19-9 and TSR delivered the second best prognostication after mGPS. Furthermore, CMS4 showed association with TSR and several laboratory markers (albumin and platelet derived factors), but not with other SIR descriptors. CMS did not show any association with CEA and CA19-9 tumor markers.

**Conclusion:** More routinely available TME, SIR and tumor markers alone and in combination deliver reliable prognostic data for choosing the patients with higher risk for propagation. CMS4 is linked with high TSR and poor prognosis, but in overall, CMS-classification showed only limited effect on SIR- and tumor-markers.

## Introduction

Colorectal cancer (CRC) is amongst the most frequent malignancies worldwide, and the second most common cause of death in cancer patients [1]. Despite advancements in targeted therapy, a large subset of CRC patients is not eligible for specialized treatment or often presents resistance [2].

The tumor microenvironment (TME) is an inseparable element of malignancies, providing comprehensive understanding to cancer biology and presenting strong prognosticators of patient outcome and therapy response [3–5]. There are several aspects of the TME in CRC, that provide clinically relevant information on tumor biology and patient outcome, moreover, some of these can be assessed conveniently by hematoxylin-eosin (HE) slides. Such markers may focus on the inflammatory infiltrate, like the Klintrup-Makinen (KM) score, that describes the inflammatory infiltrate at the invasive front without subtle measurements and is reported to be positively linked with favorable prognosis [6, 7]. Another set of TME markers is based on the assessment of stromal matrix and its components, like the tumor-stroma ratio (TSR) [8]. The TSR provides information on the amount of stromal content at the invasive front of the tumor [9]. Stroma-high (or TSR–high) tumors are linked to a more aggressive phenotype with notably poor prognosis and potential resistance to standard chemotherapy [10, 11]. Furthermore, utilizing the combination of these two, a novel grading system, the so-called Glasgow Microenvironment Score (GMS), was introduced [12]. The GMS, a combined assessment of stromal and inflammatory infiltrate in CRC, further improves the risk stratification of CRC patients and is also a strong independent prognostic factor [13].

To further understand the complex biological behavior of CRC, the consensus molecular subtyping consortium identified four distinct subclasses of CRC with the help of transcriptome-based gene expression pattern analysis, the consensus molecular subtypes (CMS) [14]. The TME has particular role in the CMS1 (also known as “immune”) and the CMS4 (a.k.a. “mesenchymal”) subtype [15]. CMS1 tumors exhibit abundant antitumoral inflammatory infiltrate and overexpression of genes associated with CD8^+^, T helper1 cell activation and T cell attracting chemokines [15], and are enriched in mismatch repair deficient (dMMR) tumors, while the mesenchymal subtype displays pronounced stromal infiltration and TGFβ signaling [14, 16]. Although currently present in the research field only, CMS classification seems a promising risk stratification method and soon might play an important role in both predicting response to traditional agents and personalized therapy as well, since the usual targeted agents may be particularly ineffective in CMS4 tumors [17, 18].

Markers of systemic inflammatory reaction (SIR), like C reactive protein (CRP), absolute neutrophil count (ANC) or lymphocyte count (ALC), or platelet count (APC), are often associated with poor prognosis in many cancer types, including CRC [19, 20]. Several inflammation-related markers might be associated with TME, however, their relationship to CMS, or more specifically, CMS4, is yet to be explored [21]. Some components of the SIR can be examined in combination, which might be utilized using composite ratios or cumulative scores. The modified Glasgow Prognostic Score (mGPS), comprising of serum albumin and CRP, is an approved indicator of systemic inflammatory processes, as well as cachexia [22, 23]. In patients with CRC, mGPS accurately predicts outcomes, with a high score associated with worse overall survival (OS) [22]. Neutrophil-lymphocyte ratio (NLR) and platelet-lymphocyte ratio (PLR), and neutrophil-platelet score (NPS) are also robust inflammation-related prognosticators in many cancers, including CRC [21, 23, 24].

Besides the TME, and the SIR, other readily available blood markers may also reflect tumor aggressiveness and predict patient outcome. Carcinoembryonic antigen (CEA) and carbohydrate antigen 19-9 (CA19-9) are frequently used tumor markers in CRC follow-up, and while these might not have the same efficacy in monitoring recurrence [25], they might be useful in certain subgroups of CRC patients [26, 27]. Even though these markers are not suited for screening purposes, their elevated levels are associated with advanced stages and therefore worse outcomes, or even therapy resistance [28–30]. Currently it is unclear, how these tumor markers are associated with the tumor microenvironment, SIR or CMS.

The aim of our study was to investigate the relation between microenvironment, systemic inflammation, CMS and tumor markers, as well as to explore and compare their prognostic significance. We investigated a few markers of systemic inflammation, including ANC, ALC, APC, CRP, albumin, and derived scores, like mGPS, NLR and to examine their relation to TME markers, tumor markers and CMS.

## Methods

### Patients and clinical data collection

185 stage I-IV patients, who underwent surgery due to colorectal cancer between 2009–2017, were selected retrospectively and their slides and formaline fixed paraffin embedded (FFPE) blocks were retrieved from the archives of the Department of Pathology and Experimental Cancer Research, Semmelweis University, Budapest, Hungary. Patients who received neoadjuvant treatment, died within 30 days of surgery, had other synchronous or metachronous primary colorectal cancer, or another malignancy requiring systemic treatment in their history, were excluded from the study. Patient history and laboratory results were collected using the medical database of Semmelweis University (MedSolution, T-Systems, Budapest, Hungary). Preoperative serum CA19-9 and CEA levels were measured routinely using Abbott Architect CA 19-9 XR immunoassay (Chicago, IL, United States of America) and Abbott Architect CEA immunoassay (Chicago, IL, United States of America). Pathological characterization of surgically resected primary tumors was performed according to the UICC TNM classification, 8^th^ edition.

### Assessment of tumor microenvironment

**FIGURE 1:**
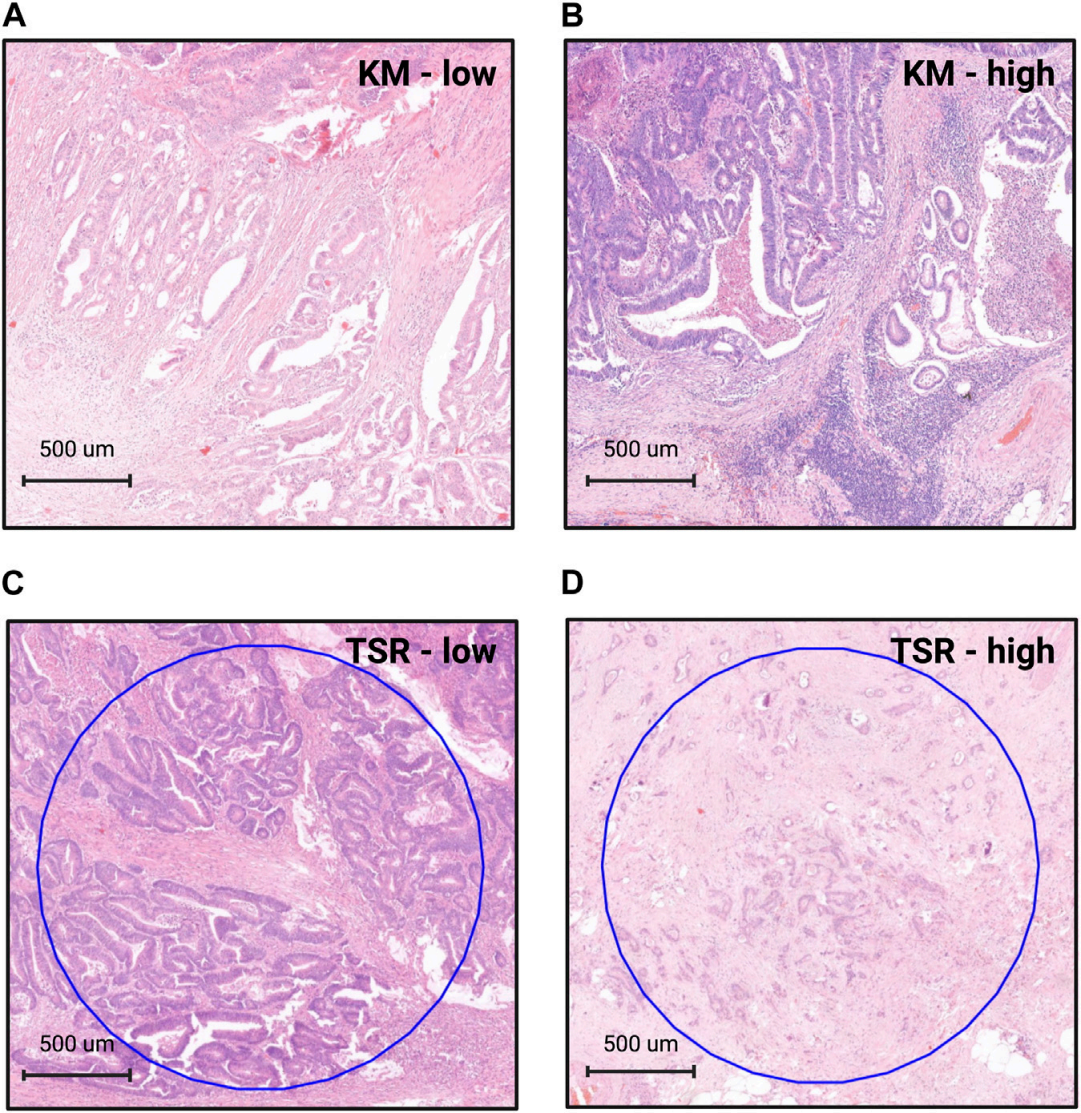
Grading the tumor microenvironment on HE-slides. For assessing Klintrup-Makinen grade, the inflammatory infiltrate has to be examined at the invasive front of the tumor. In case there is none or only patchy, mild inflammation, cases are classified as KM-low **(A)**. If there is a band- or cup-like, florid inflammatory infiltrate with destruction of tumor cells, cases are graded as KM-high **(B)**. When assessing the tumor-stroma ratio (TSR), the stromal hotspot has to be evaluated at the invasive front using a ×10 objective. Tumor cells must be present at all four poles of the field of view. If stromal content is less than 50% of the examined area, cases are graded as TSR-low **(C)**. If stromal content equals or exceeds 50% of this area, cases are graded as TSR-high **(D)**. List of abbreviations: HE, hematoxylin-eosin; KM, Klintrup-Makinen grade; TSR, tumor-stroma ratio. Figure was created using https://www.biorender.com/.

TSR was evaluated on HE-stained slides in accordance with recommendations [8]: stromal content was estimated visually per 10% increments at ×10 magnification field of the invasive front of the tumor (containing the stromal hotspot). If stromal content exceeded 50% of the examined area, patients were classified as TSR-high, if not, they were categorized as TSR-low (Figure 1).

KM grade was estimated according to the criteria established by Klintrup et al [6]. Briefly, inflammatory reaction was graded as KM-low when there was no or only mild increase in inflammatory cells at the invasive front, and a KM-high score was given when there was a band- or cup-like infiltrate of inflammatory cells at the margin with destruction of cancer cells (Figure 1). The GMS is a combination of KM score and TSR. Briefly, when there was extensive inflammatory reaction (KM-high), a score of GMS0 was given. In case of stroma-low and KM-low tumors, a score of GMS1, and in case of stroma-high and KM-low tumors a score of GMS2 was given (Supplementary Table S1), as described previously [12, 13]. All parameters were graded by two independent observers (AJ and TSM) blinded to clinicopathological data and disease outcome.

### Assessment of SIR and tumor markers

All blood samples were obtained from patients within 30 days before surgery. Cutoff values and scoring of SIR markers are included in Supplementary Table S1.

### Microarray construction and immunohistochemistry

Tissue microarray (TMA) blocks containing 6 × 9 cores (core diameter: 2 mm) selected from surgically derived FFPE blocks of 167 patients were created using TMA Master1000 (3DHistech, Budapest, Hungary). At least two representative cores were selected per case. Non-neoplastic kidney samples were used as normal tissues and stain controls in each block.

Immunohistochemistry was performed on 4 um thick sections of TMAs. For mismatch repair status (MMR) assessment, anti-MLH1, anti-PMS2, anti-MSH2, and anti-MSH6 primary stains were used and graded as recommended [31]. CMS classification was carried out on proficient MMR (pMMR) samples using anti-cytokeratin (CK), anti-CDX2, anti-FRMD6, and anti-ZEB1 immunhistochemistry as described by Ten Hoorn et al [32]. For further details of immunohistochemistry, please see Supplementary Table S2. Cytoplasmic CK and FRMD6, nuclear CDX2, as well as membrane and cytoplasmic HTR2B were graded as low, moderate or high. In case of ZEB1, the presence of nuclear staining was scored as either present or absent. All CMS stains were graded both in accordance with Ten Hoorn et al’s recommendations, and, apart from ZEB1, using H-score as well [33].

All immunohistochemical reactions were assessed by two observers (AJ and TSM).

### CMS classification

**FIGURE 2:**
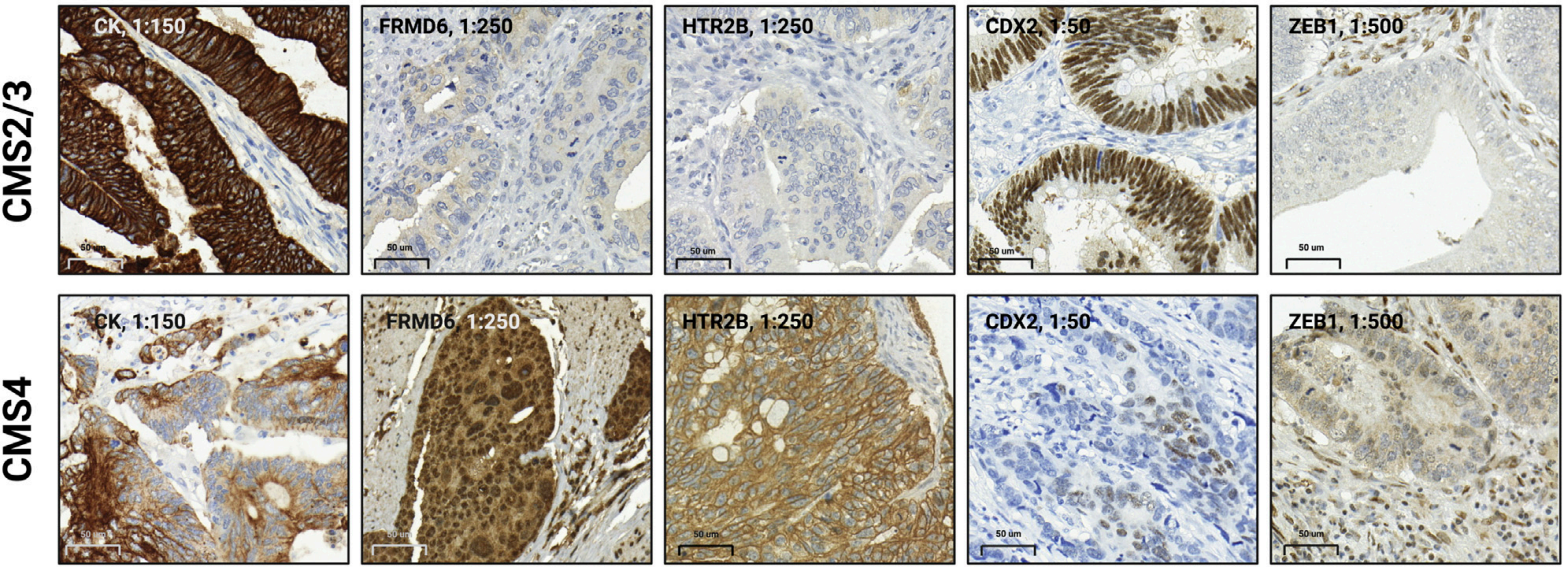
Immunohistochemistry-based phenotype of epithelial and mesenchymal subtypes of colorectal cancer (CRC). In CMS2/3 or epithelial CRCs, predominantly strong positive CK reaction, negative or weak positive FRMD6 and HTR2B stains, prominent CDX2 nuclear reaction and lack of nuclear ZEB1 expression can be observed. In CMS4 or mesenchymal tumors, usually a somewhat weaker or less intense CK reaction, strong positive FRMD6 and HTR2B reaction, loss of CDX2 stain, and presence of nuclear ZEB1 expression can be detected. CMS classification was carried out using an online, validated CMS-classifier at https://crcclassifier.shinyapps.io/appTesting/. Figure was created using https://www.biorender.com/.

Cases with deficient MMR-status (dMMR) were classified as CMS1. For proficient MMR tumors, CMS classification was performed using an online, TMA-based and validated, robust and reliable random forest classifier^1^ as described previously [32, 33]. Briefly, four immunostains (ZEB1, HTRB2, FRMD6 and CDX2) were selected based on distinct gene expression profile differences in CMS2/3 and CMS4 tumors. In pMMR tumors, the stain intensity and content of these four stains in tumor epithelium correlates with their CMSs [32]. Typically, low FRMD6 and HTR2B staining intensities, lack of nuclear ZEB1 expression and strong CDX2 stain correlate with epithelial subtypes (CMS2/3); while strong positive FRMD6 and HTR2B, loss of CDX2 and positive nuclear ZEB1 reaction is expected in mesenchymal CRCs (CMS4) (Figure 2) [33]. In case the probability of a CMS was estimated higher than 0.6, we automatically labeled the case in concordance with the software. Where the probability of estimated CMS was between 0.5 and. 0.6, the case was automatically excluded from our analysis. In total, 12 cases were excluded due to uncertain subtyping.

### Establishing a novel scoring system: stroma-tumor marker (STM) score

To reflect the biological behaviour of certain cancer subtypes classified by TSR and CA19-9, a novel scoring system was established by combining CA19-9 and TSR into stroma-tumor marker (STM) score. In case of TSR-low and CA 19-9 low cases, STM 0 score was given. If either CA19-9 or TSR was classified as high, but the other marker as low, an STM 1 score was given. When both markers were classified as “high,” STM 2 score was given (Supplementary Table S1).

### Statistical analysis

Shapiro-Wilks test of normality was performed on the continuous variables. In order to determine the relationship between clinicopathological features and categorical variables, Chi-squared test was performed. Mann-Whitney *U*-test or Kruskal-Wallis H-test was performed to examine the correlation between clinicopathological features and continuous variables. Spearman’s rank order correlation coefficent was calculated to investigate the correlation between certain systemic inflammation-related and tumor markers. The relationship between TME, systemic and tumor markers and survival was carried out using Kaplan-Meier log-rank survival analysis. To assess how certain variables affect the overall survival (OS), uni- and multivariate Cox regression analysis was performed. All variables that reached *p* < 0.1 in the univariate analysis were included for the multivariate analysis. The complete statistical analysis was performed using SPSS version 28.0.1.0 (IBM, Armonk, NY, United States).

## Results

### Patient characteristics

Altogether, 185 patients were included in our cohort, 155 patients had available CEA, and 135 patients had available CA19-9 results. CMS classification was carried out in 155 patients. Further information can be found in Tables 1–3 and Supplementary Table S1.

**TABLE 1:** The relationship between tumor-stroma ratio (TSR), Klintrup-Makinen (KM) grade and clinicopathological parameters.

	All patients (n = 185^*^)	TSR (n = 185)			KM grade (n = 185)		
		TSR-low (n = 121)	TSR-high (n = 64)	*p*-value	KM-low (n = 123)	KM-high (n = 62)	*p*-value
Age (n = 185)				***p* = 0.022**			*p* = 0.237
<65	65 (35%)	**34 (28%)**	**31 (48%)**		45 (37%)	20 (32%)	
65-74	77 (42%)	**56 (46%)**	**21 (33%)**		54 (44%)	23 (37%)	
75<	43 (23%)	**31 (26%)**	**12 (19%)**		24 (20%)	19 (31%)	
Sex (n = 185)				*p* = 0.353			*p* = 0.434
Female	97 (52%)	60 (50%)	37 (58%)		67 (55%)	30 (48%)	
Male	88 (48%)	61 (50%)	27 (42%)		56 (46%)	32 (52%)	
Location (n = 185)				*p* = 0.350			*p* = 0.488
Right colon	75 (41%)	53 (44%)	22 (34%)		47 (38%)	28 (45%)	
Left colon	58 (31%)	34 (28%)	24 (37.5%)		42 (34%)	16 (26%)	
Rectum	52 (28%)	34 (28%)	18 (28%)		35 (28%)	18 (29%)	
pT (n = 185)				***p* = 0.043**			**p = 0.029**
pT1	2 (1%)	**2 (2%)**	**0 (0%)**		**0 (0%)**	**2 (3%)**	
pT2	34 (18%)	**28 (23%)**	**6 (9%)**		**22 (18%)**	**12 (19%)**	
pT3	134 (72%)	**84 (69%)**	**50 (78%)**		**87 (71%)**	**47 (76%)**	
pT4	15 (8%)	**7 (6%)**	**8 (13%)**		**14 (11%)**	**1 (2%)**	
pN (n = 184)				***p* < 0.001**			**p = 0.024**
pN0	82 (45%)	**66 (55%)**	**16 (25%)**		**47 (39%)**	**35 (57%)**	
pN1	67 (36%)	**37 (31%)**	**30 (47%)**		**46 (38%)**	**21 (34%)**	
pN2	35 (19%)	**17 (14%)**	**18 (28%)**		**29 (24%)**	**6 (10%)**	
M (n = 185)				***p* < 0.001**			**p = 0.009**
M0	143 (77%)	**104 (86%)**	**39 (61%)**		**88 (72%)**	**55 (89%)**	
M1	42 (23%)	**17 (14%)**	**25 (39%)**		**35 (29%)**	**7 (11%)**	
Stage (n = 185)				***p* < 0.001**			**p = 0.017**
I	26 (14%)	**23 (19%)**	**3 (5%)**		**16 (13%)**	**10 (16%)**	
II	48 (26%)	**37 (31%)**	**11 (17%)**		**25 (20%)**	**23 (37%)**	
III	69 (37%)	**44 (36%)**	**25 (39%)**		**47 (38%)**	**22 (36%)**	
IV	42 (23%)	**17 (14%)**	**25 (39%)**		**35 (29%)**	**7 (11%)**	
Grade (n = 185)				*p* = 0.108			*p* = 0.629
Low/moderate	161 (87%)	109 (90%)	52 (81%)		106 (86%)	55 (89%)	
High	24 (13%)	12 (10%)	12 (19%)		17 (14%)	7 (11%)	
Lymphatic invasion (n = 185)				***p* < 0.001**			*p = 0.093*
Not present	122 (66%)	**94 (78%)**	**28 (44%)**		*76 (62%)*	*46 (74%)*	
Present	63 (34%)	**27 (22%)**	**36 (56%)**		*47 (38%)*	*16 (26%)*	
Perineural invasion (n = 185)				***p* = 0.002**			*p* = 0.247
Not present	170 (92%)	**117 (97%)**	**53 (83%)**		111 (90%)	59 (95%)	
Present	15 (8%)	**4 (3%)**	**11 (17%)**		12 (10%)	3 (5%)	
mGPS (n = 96)				*p* = 0.395			*p* = 0.680
mGPS 0	39 (41%)	28 (44%)	11 (34%)		27 (40%)	14 (50%)	
mGPS 1	36 (38%)	24 (38%)	12 (38%)		25 (37%)	9 (32%)	
mGPS 2	21 (22%)	12 (20%)	9 (28%)		15 (22%)	5 (18%)	
CMS (n = 155)				*p = 0.054*			*p* = 0.354
CMS1	16 (10%)	*12 (12%)*	*4 (7%)*		8 (8%)	8 (15%)	
CMS2/3	109 (70%)	*75 (74%)*	*34 (63%)*		73 (72%)	36 (68%)	
CMS4	30 (19%)	*14 (14%)*	*16 (30%)*		21 (21%)	9 (17%)	
CEA (n = 155)				***p* = 0.029**			*p* = 0.919
CEA-low	101 (65%)	**72 (71%)**	**29 (54%)**		70 (65%)	31 (65%)	
CEA-high	54 (35%)	**29 (29%)**	**25 (46%)**		37 (35%)	17 (35%)	
CA19-9 (n = 135)				***p* = 0.035**			*p* = 0.584
CA19-9-low	111 (82%)	**80 (87%)**	**31 (72%)**		77 (81%)	34 (85%)	
CA19-9-high	24 (18%)	**31 (13%)**	**28 (28%)**		19 (19%)	6 (15%)	

The relationship between TSR, KM, grade and clinicopathological features was assessed using Chi-squared test. Significant correlations were marked with bold font, while tendencies where *p* < 0.1 were marked with italic font. In some cases, percentages do not add up to 100% precisely due to rounding. Abbreviations: TSR, tumor-stroma ratio; KM, grade, Klintrup Makinen grade; mGPS, modified Glasgow Prognostic Score; CMS, consensus molecular subtypes; CEA, carcinoembryonic antigen; CA 19-9, Carbohydrate antigen 19-9.

**TABLE 2:** The relationship between Glasgow microenvironment score (GMS) and clinicopathological parameters.

Clinico-pathological features	All patients (n = 185^*^)	GMS (n = 185)			
		GMS0 (n = 102)	GMS1 (n = 42)	GMS2 (n = 41)	*p*-value
Age (n = 185)					***p* = 0.008**
<65	65 (35%)	**26 (26%)**	**16 (38%)**	**23 (56%)**	
65-74	77 (42%)	**50 (49%)**	**14 (33%)**	**13 (32%)**	
75<	43 (23%)	**26 (26%)**	**12 (29%)**	**5 (12%)**	
Sex (n = 185)					*p* = 0.904
Female	97 (52%)	52 (51%)	23 (55%)	22 (54%)	
Male	88 (48%)	50 (49%)	19 (45%)	19 (46%)	
Location (n = 185)					*p* = 0.158
Right colon	75 (41%)	49 (48%)	13 (31%)	13 (32%)	
Left colon	58 (31%)	25 (25%)	17 (41%)	16 (39%)	
Rectum	52 (28%)	28 (28%)	12 (29%)	12 (29%)	
pT (n = 185)					***p* = 0.015**
pT1	2 (1%)	**2 (2%)**	**0 (0%)**	**0 (0%)**	
pT2	34 (18%)	**26 (26%)**	**6 (14%)**	**2 (5%)**	
pT3	134 (72%)	**70 (69%)**	**32 (76%)**	**32 (78%)**	
pT4	15 (8%)	**4 (4%)**	**4 (10%)**	**7 (17%)**	
pN (n = 184)					***p* = 0.001**
pN0	82 (45%)	**56 (55%)**	**19 (46%)**	**7 (17%)**	
pN1	67 (36%)	**32 (31%)**	**14 (34%)**	**21 (51%)**	
pN2	35 (19%)	**14 (14%)**	**8 (20%)**	**13 (32%)**	
M (n = 185)					***p* < 0.001**
M0	143 (77%)	**89 (87%)**	**32 (76%)**	**22 (54%)**	
M1	42 (23%)	**13 (13%)**	**10 (24%)**	**19 (46%)**	
Stage (n = 185)					***p* < 0.001**
I	26 (14%)	**21 (21%)**	**4 (10%)**	**1 (2%)**	
II	48 (26%)	**30 (29%)**	**13 (31%)**	**5 (12%)**	
III	69 (37%)	**38 (37%)**	**15 (36%)**	**16 (39%)**	
IV	42 (23%)	**13 (13%)**	**10 (24%)**	**19 (46%)**	
Grade (n = 185)					*p* = 0.718
Low/moderate	161 (87%)	90 (88%)	35 (83%)	36 (88%)	
High	24 (13%)	12 (12%)	7 (17%)	5 (12%)	
Lymphatic invasion (n = 185)					***p* < 0.001**
Not present	122 (66%)	**74 (73%)**	**32 (76%)**	**16 (39%)**	
Present	63 (34%)	**28 (28%)**	**10 (24%)**	**25 (61%)**	
Perineural invasion (n = 185)					***p* = 0.010**
Not present	170 (92%)	**97 (95%)**	**40 (95%)**	**33 (81%)**	
Present	15 (8%)	**5 (5%)**	**2 (5%)**	**8 (20%)**	
mGPS (n = 96)					*p* = 0.316
mGPS 0	39 (41%)	25 (52%)	7 (33%)	9 (35%)	
mGPS 1	36 (38%)	16 (33%)	7 (33%)	11 (42%)	
mGPS 2	21 (22%)	7 (15%)	7 (33%)	6 (23%)	
CMS (n = 155)					*p* = 0.119
CMS1	16 (10%)	12 (14%)	1 (3%)	3 (8%)	
CMS2/3	109 (70%)	59 (69%)	28 (82%)	22 (61%)	
CMS4	30 (19%)	14 (17%)	5 (15%)	11 (37%)	
CEA (n = 155)					*p* = 0.215
CEA-low	101 (65%)	55 (68%)	25 (71%)	21 (54%)	
CEA-high	54 (35%)	26 (32%)	10 (29%)	18 (46%)	
CA19-9 (n = 135)					***p* = 0.011**
CA19-9-low	111 (82%)	**62 (86%)**	**29 (91%)**	**20 (65%)**	
CA19-9-high	24 (18%)	**10 (14%)**	**3 (9%)**	**11 (36%)**	

The relationship between the Glasgow microenvironment score and clinicopathological features was assessed using Chi-squared test. Significant correlations were marked with bold font, while tendencies where *p* < 0.1 were marked with italic font. In some cases, percentages do not add up to 100% precisely due to rounding. Abbreviations: GMS, Glasgow microenvironment score; mGPS, modified Glasgow Prognostic Score; CMS, consensus molecular subtypes; CEA, carcinoembryonic antigen; CA 19-9, Carbohydrate antigen 19-9.

**TABLE 3:** The relationship between consensus molceular subtypes and clinicopathological parameters.

Clinico-pathological features	All patients (n = 185^*^)	CMS (n = 155)			
		CMS1 (n = 16)	CMS2/3 (n = 109)	CMS4 (n = 30)	*p*-value
Age (n = 185)					*p* = 0.883
<65	65 (35%)	4 (25%)	35 (32%)	12 (40%)	
65-74	77 (42%)	8 (50%)	49 (45%)	12 (40%)	
75<	43 (23%)	4 (25%)	25 (23%)	6 (20%)	
Sex (n = 185)					*p* = 0.337
Female	97 (52%)	6 (38%)	59 (54%)	18 (60%)	
Male	88 (48%)	10 (63%)	50 (46%)	12 (40%)	
Location (n = 185)					***p* = 0.006**
Right colon	75 (41%)	**13 (81%)**	**37 (34%)**	**13 (43%)**	
Left colon	58 (31%)	**2 (13%)**	**36 (33%)**	**11 (37%)**	
Rectum	52 (28%)	**1 (6%)**	**36 (33%)**	**6 (20%)**	
pT (n = 185)					*p = 0.062*
pT1	2 (1%)	0 (0%)	2 (2%)	0 (0%)	
pT2	34 (18%)	3 (19%)	22 (20%)	1 (3%)	
pT3	134 (72%)	11 (69%)	80 (73%)	23 (77%)	
pT4	15 (8%)	2 (13%)	5 (5%)	6 (20%)	
pN (n = 184)					***p* = 0.001**
pN0	82 (45%)	**6 (38%)**	**62 (57%)**	**6 (20%)**	
pN1	67 (36%)	**4 (25%)**	**32 (30%)**	**13 (43%)**	
pN2	35 (19%)	**6 (38%)**	**14 (13%)**	**11 (37%)**	
M (n = 185)					***p* = 0.022**
M0	143 (77%)	**15 (94%)**	**86 (79%)**	**18 (60%)**	
M1	42 (23%)	**1 (6%)**	**23 (21%)**	**12 (40%)**	
Stage (n = 185)					***p* = 0.006**
I	26 (14%)	**2 (13%)**	**9 (17%)**	**0 (0%)**	
II	48 (26%)	**4 (25%)**	**37 (34%)**	**5 (17%)**	
III	69 (37%)	**9 (56%)**	**30 (28%)**	**13 (43%)**	
IV	42 (23%)	**1 (6%)**	**23 (21%)**	**12 (40%)**	
Grade (n = 185)					***p* < 0.001**
Low/moderate	161 (87%)	**9 (56%)**	**100 (92%)**	**24 (80%)**	
High	24 (13%)	**7 (44%)**	**9 (8%)**	**6 (20%)**	
Lymphatic invasion (n = 185)					***p* < 0.001**
Not present	122 (66%)	**9 (56%)**	**83 (86%)**	**12 (40%)**	
Present	63 (34%)	**7 (44%)**	**26 (24%)**	**18 (60%)**	
Perineural invasion (n = 185)					***p* = 0.006**
Not present	170 (92%)	**14 (88%)**	**104 (95%)**	**26 (77%)**	
Present	15 (8%)	**2 (13%)**	**5 (5%)**	**7 (23%)**	
mGPS (n = 96)					*p* = 0.486
mGPS 0	39 (41%)	2 (40%)	24 (44%)	10 (56%)	
mGPS 1	36 (38%)	3 (60%)	17 (31%)	5 (28%)	
mGPS 2	21 (22%)	0 (0%)	14 (26%)	3 (17%)	
CEA (n = 155)					*p* = 0.439
CEA-low	101 (65%)	7 (58%)	62 (70%)	15 (58%)	
CEA-high	54 (35%)	4 (42%)	27 (30%)	11 (42%)	
CA19-9 (n = 135)					*p* = 0.215
CA19-9-low	111 (82%)	12 (100%)	63 (84%)	17 (77%)	
CA19-9-high	24 (18%)	0 (0%)	12 (16%)	5 (23%)	

The relationship between the consensus molecular subtypes and clinicopathological features was assessed using Chi-squared test. Significant correlations were marked with bold font, while tendencies where *p* < 0.1 were marked with italic font. In some cases, percentages do not add up to 100% precisely due to rounding. Abbreviations: CMS, consensus molecular subtypes; mGPS, modified Glasgow Prognostic Score; CEA, carcinoembryonic antigen; CA 19-9, Carbohydrate antigen 19-9.

### TME characteristics

Patients with TSR-high tumors were significantly associated with higher pT (*p* = 0.043), pN (*p* < 0.001) and M (*p* < 0.001) descriptors (Table 1). Also, lymphatic and perineural invasion was significantly higher amongst TSR-high patients (*p* < 0.001 and *p* = 0.002) (Table 1). TSR correlated with age, CEA and CA19-9, using Chi-squared test (*p* = 0.022, *p* = 0.029, *p* = 0.035). Similarly, KM-low correlated with advanced pT, pN, stage and M-status (*p* = 0.029, *p* = 0.024, *p* = 0.017 and *p* = 0.009) and also, a tendency towards lymphatic invasion (*p* = 0.093) was found (Table 1). As expected based on TSR and KM grading results, GMS was also associated with more advanced pT, pN and M descriptors and stage (*p* = 0.015, *p* = 0.001, *p* < 0.001 and *p* < 0.001) and lymphatic and perineural invasion (*p* < 0.001, *p* = 0.010), and there was a tendency towards vascular invasion (*p* = 0.065) (Table 2). KM, TSR or GMS were not associated with any SIR markers (Supplementary Tables S3, S4).

### Pre-operative SIR assessment

**FIGURE 3:**
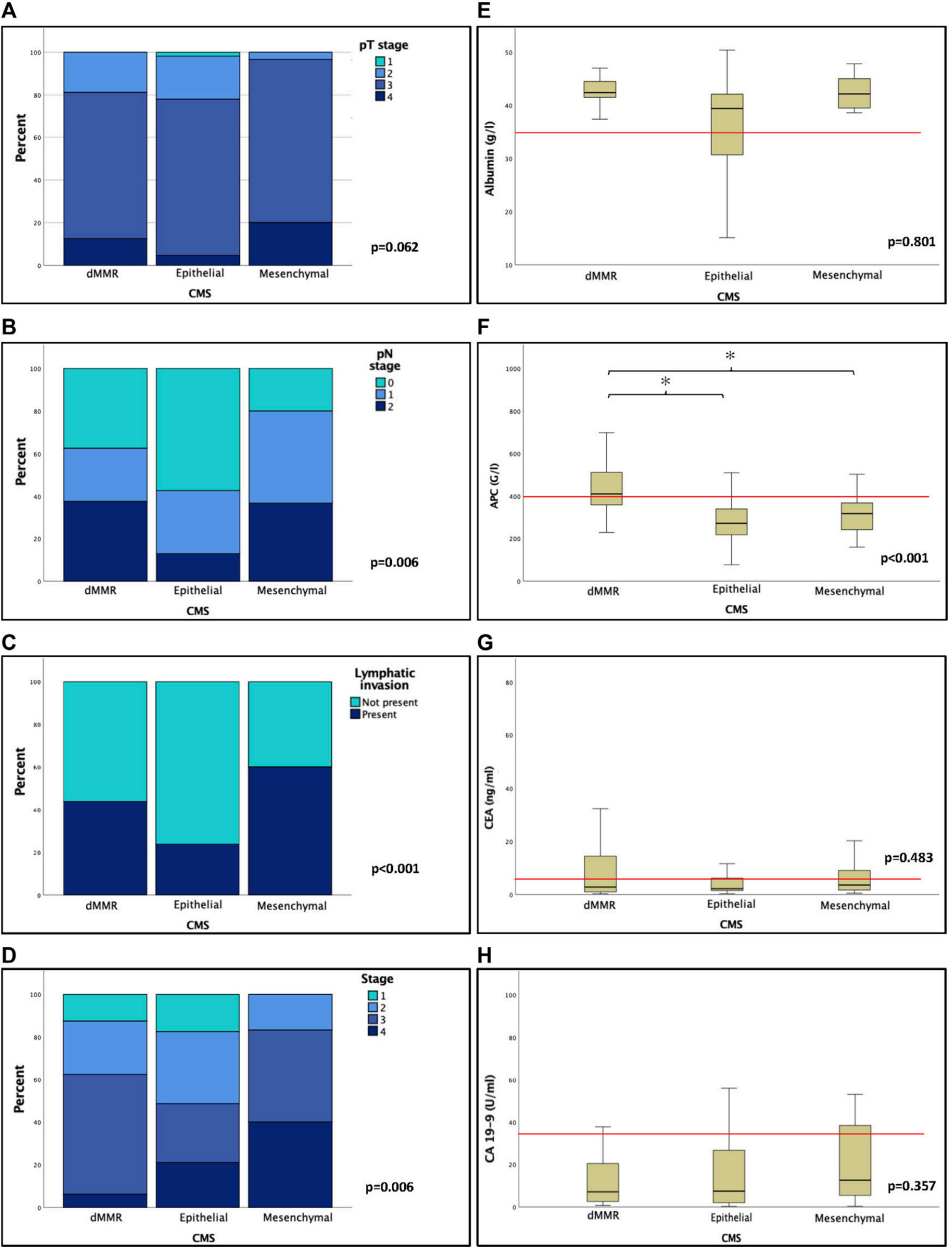
The relationship of CMS and clinicopathological features was analysed using Chi-square test **(A–D)**. Generally, mesenchymal subtype was associated with higher T, N, and TNM status and presence of lymphatic invasion. Markers of systemic inflammation and tumor markers as well as their association with CMS were also examined using non-parametric Kruskal-Wallis tests. Preoperative absolute platelet count was significantly elevated in dMMR tumors compared to both epithelial and mesenchymal subtypes. The relationship of CMS and SIR and tumor markers was assessed using non-parametric Mann-Whitney and Kruskal-Wallis test **(E–H)**. There was a significant association towards elevated APC in dMMR tumors. Tumor markers were not significantly associated with CMS. Abbreviations: dMMR, Mismatch repair deficient/deficiency; CMS, consensus molecular subtypes; APC, absolute platelet count; CEA, carcinoembryonic antigen; CA 19-9, carbohydrate antigen 19-9. Asterisks (*) mark significant associations *p* < 0.05.

Elevation of serum CRP was associated with increasing stage (*p* = 0.002), pT (*p* < 0.001), distant metastasis (*p* = 0.007), higher grade (*p* = 0.027), vascular invasion (*p* = 0.026), lymphatic invasion (*p* = 0.032) and there was a trend towards perineural invasion (*p* = 0.063) (Supplementary Table S3). There was a significant correlation between ANC and higher pT (*p* = 0.011) and a trend towards advanced pN (*p* = 0.058) (Supplementary Table S3). ALC did not correlate with any of the examined features, but with younger age (*p* = 0.007). APC was significantly elevated in males (*p* = 0.003), associated with right-sidedness (*p* = 0.013), CMS1 (*p* < 0.001), showed significant association with lymphatic invasion (*p* = 0.045) and there was a tendency towards distant metastasis (*p* = 0.068) and vascular invasion (*p* = 0.077) (Supplementary Table S3, Figure 3).

NLR did not show any correlation with tumor descriptors, but PLR showed significant association with higher age (*p* = 0.046), right sidedness (*p* = 0.022) and CMS1 (*p* = 0.005) using nonparametric Kruskal-Wallis H-test (Supplementary Table S3). The mGPS showed significant association with higher grade (*p* = 0.042) and a tendency towards elevated pT (*p* = 0.057) (Supplementary Table S4). NLR and PLR did not show any correlation with clinicopathological descriptors, whereas NPS was significantly associated with male gender (*p* = 0.003), higher pT and pN stages (*p* = 0.043 and *p* = 0.032) and was inversely associated with the frequency of epithelial phenotype using Chi-squared test (*p* = 0.034) (Supplementary Table S4).

### Pre-operative serum CEA and CA19-9

**FIGURE 4:**
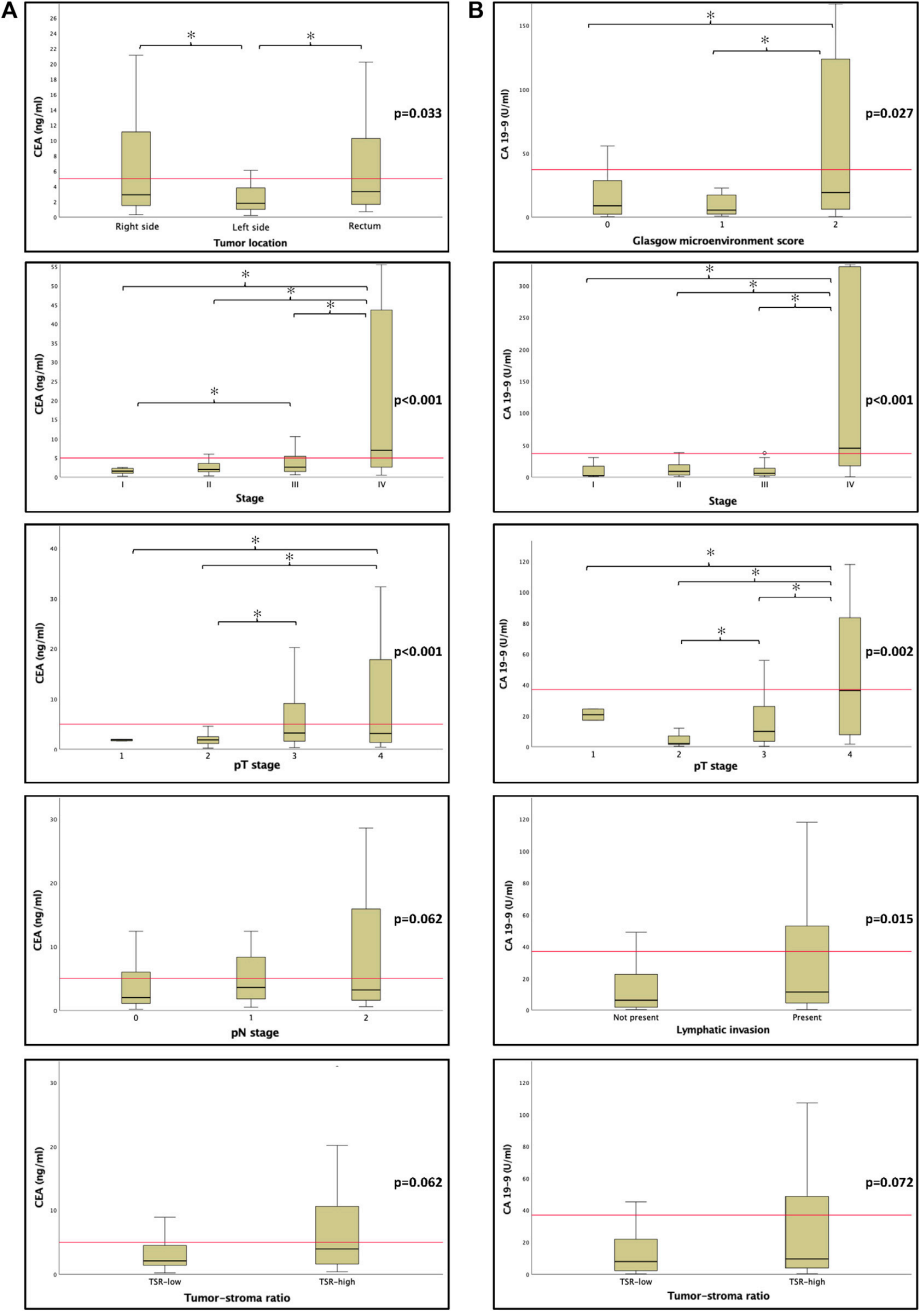
The relationship between canonical tumor markers CEA **(A)** and CA 19-9 **(B)** and clinicopathological features. Unsuprisingly, elevated tumor marker levels were mostly associated with adverse features. There was a tendency between tumor markers and high tumor-stroma ratio (TSR). The horizontal red line represents clinically relevant cut off values mentioned in Supplementary Table S1. Abbreviations: CEA, carcinoembryonic antigen; CA 19-9, carbohydrate antigen 19-9; TSR, tumor-stroma ratio. Asterisks (*) mark significant associations *p* < 0.05.

CEA was significantly lower in left sided tumors (*p* = 0.033). Elevated CEA levels were associated with stage (*p* < 0.001), pT (*p* = 0.044) and distant metastasis (*p* < 0.001) and also showed a tendency towards higher pN (*p* = 0.062) stage, besides, using Chi-squared test, TSR-high tumors were associated with higher CEA levels (*p* = 0.029) (Supplementary Table S3, Table 1 and Figure 4).

CA19-9 was also associated with stage (*p* < 0.001), pT (*p* = 0.002), distant metastasis (*p* < 0.001), lymphatic invasion (*p* = 0.015), and GMS (*p* = 0.027). There was a tendency towards vascular (*p* = 0.065) and perineural (*p* = 0.081) invasion, and with Chi-squared test there was significant association between TSR-high status and elevated CA19-9 (*p* = 0.035) (Supplementary Table S3, Table 1 and Figure 4).

### CMS immunohistochemistry and classification

Low CK expression was associated with higher age, and TSR-low (*p* = 0.012, *p* = 0.003), and inversely associated with presence of lymphatic invasion (*p* = 0.020) (Supplementary Table S5). Low FRMD6 expression was positively associated with TSR-low (*p* = 0.041), elevated serum CEA (*p* = 0.008) and albumin concentration (*p* = 0.026). Loss of CDX2 expression positively correlated with lymphatic and perineural invasion (*p* = 0.026 and *p* = 0.037), with NLR-high (*p* = 0.023) and showed tendency towards higher pT (*p* = 0.087) and increased serum albumin concentration (*p* = 0.081). The expression of ZEB1 was observed in only 14% of CMS classified cases. Interestingly, ZEB1 positive cases had lower CRP-levels (*p* = 0.039) and showed tendency towards lower mGPS (*p* = 0.074). No significant associations were revealed regarding HTR2B expression, only a tendency towards higher pN (*p* = 0.092) and M (*p* = 0.075) and higher PLR (*p* = 0.083) (Supplementary Table S5).

CMS1 was significantly associated with right colonic localization (*p* = 0.006) and higher histological grades (*p* < 0.001). CMS4 was associated with higher stage (*p* = 0.006), lymphatic and perineural invasion (*p* < 0.001 and *p* = 0.006, respectively) pN (*p* = 0.001) and M (*p* = 0.022) descriptors, and there was a tendency towards high TSR just failing to be significant (*p* = 0.054) as well (Tables 1, 3; Figure 3). We did not find significant correlation between the examined tumor markers (CEA and CA19-9) and CMS (*p* = 0.439 and *p* = 0.215) (Figure 3; Table 3).

### Survival analysis

**FIGURE 5:**
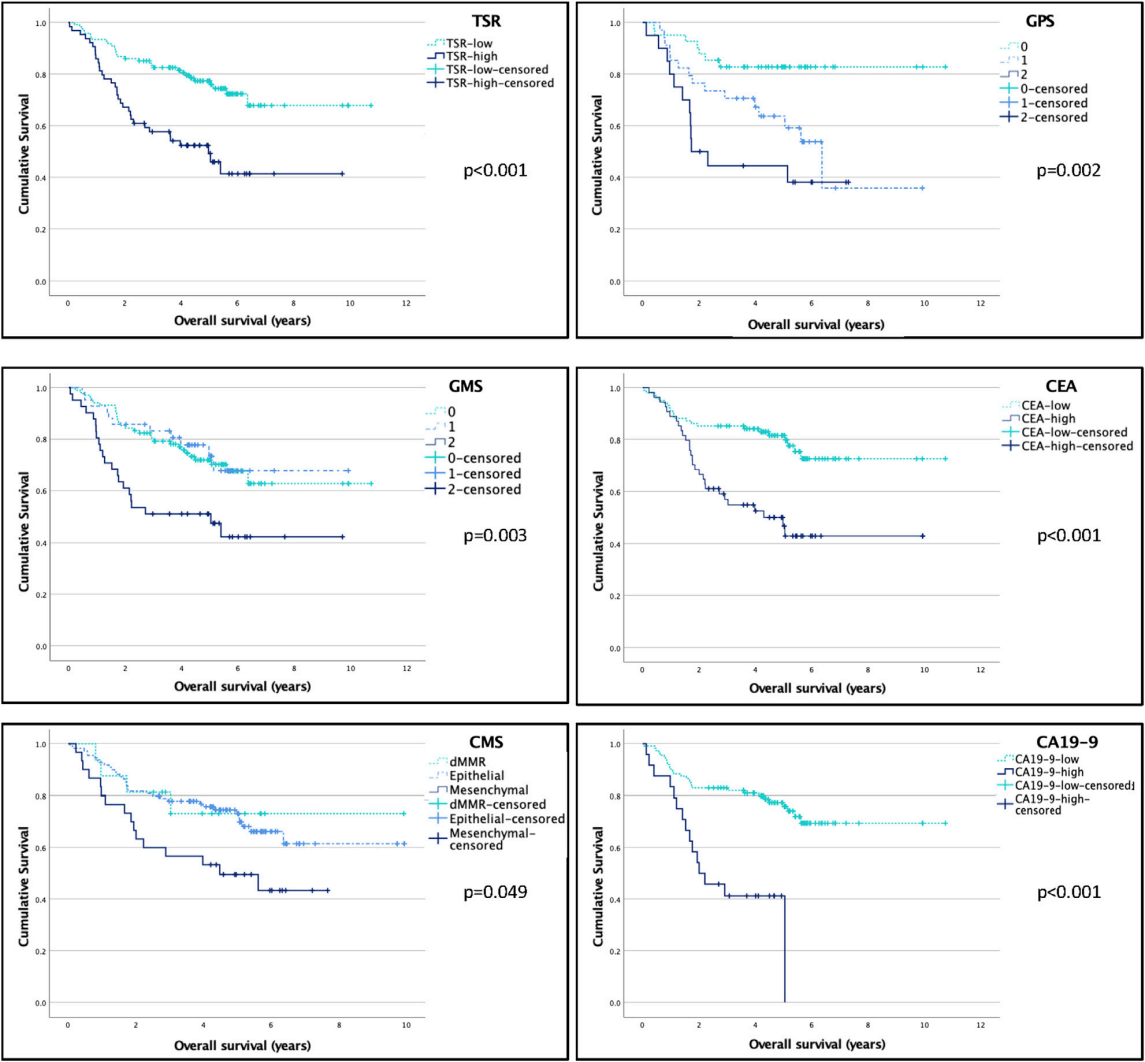
The effect of microenviromental and systemic markers on overall survival (OS). Log-rank test was used to compare the OS of certain subgroups. Bold font denotes significant correlations in the multivariate model, italic font denotes tendencies with *p* < 0.1 in the multivariate model. For further information on multivariate analysis, see Table 2. Abbreviations: TSR, tumor stroma ratio; STM score, Stroma-Tumor Marker Score; CMS, consensus molecular subtype; mGPS, modified Glasgow Prognostic Score; CEA, carcinoembryonic antigen; CA19-9, carbohydrate antigen 19-9.

With Kaplan-Meier survival analysis we found that some microenvironmental and systemic markers of CRC were associated with OS (Supplementary Table S6). Patients with high stromal content (*p* < 0.001), high GMS (*p* = 0.003), high ANC (*p* = 0.007), low albumin (*p* = 0.027), elevated CRP (*p* = 0.006), elevated CEA (*p* < 0.001) and CA19-9 (*p* < 0.001) as well as higher mGPS (*p* = 0.002) and mesenchymal subtype (*p* = 0.049) had shorter overall survival (Supplementary Table S6, Figure 5).

**FIGURE 6:**
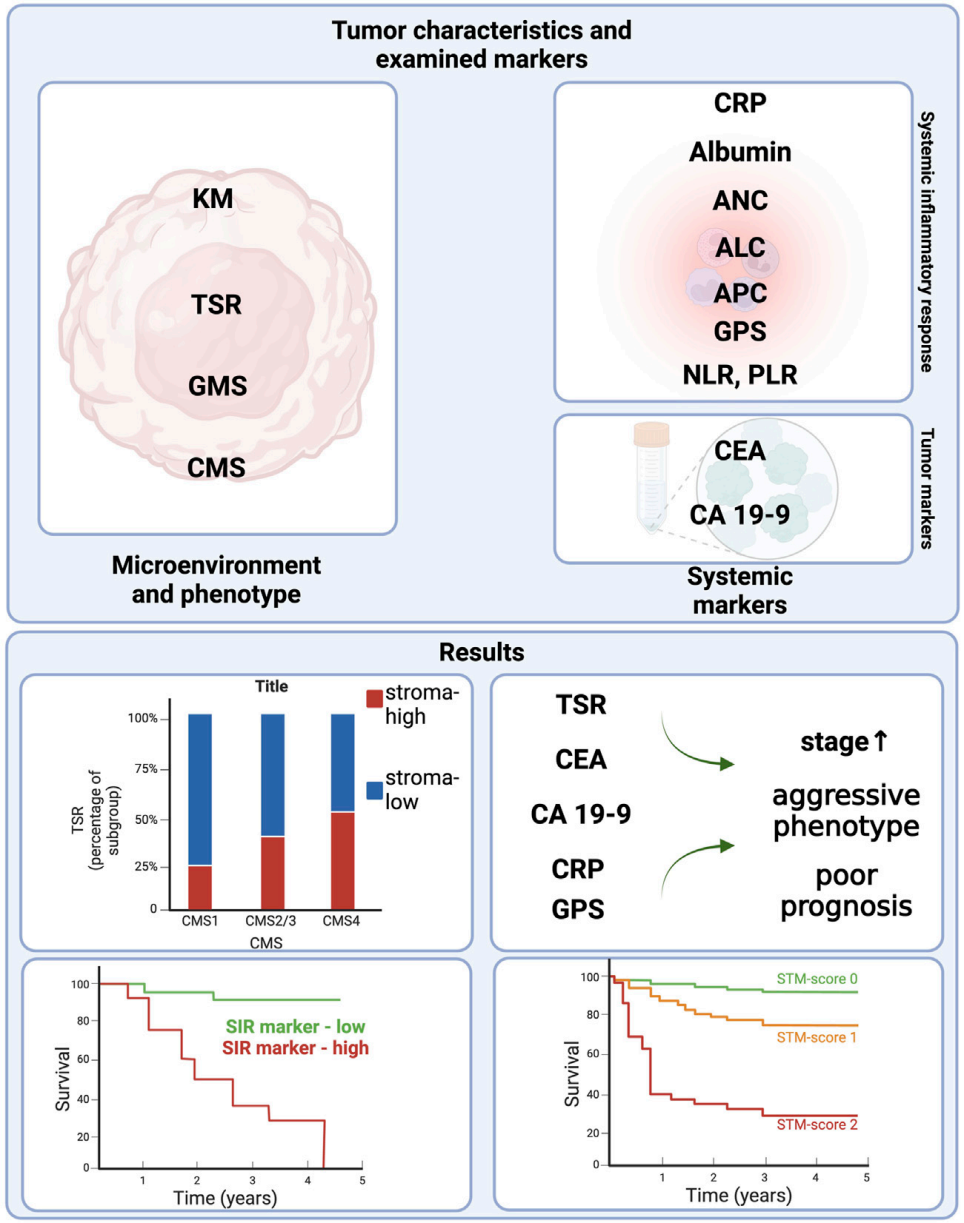
Study design and results. Figure was created using https://www.biorender.com/. Abbreviations: KM, Klintrup-Makinen grade; TSR, tumor-stroma ratio; GMS, Glasgow microenvironment score; CMS, consensus molecular subtype; CRP, C reactive protein; ANC, absolute neutrophil count; ALC, absolute lymphocyte count; APC, absolute platelet count; GPS, (modified) Glasgow Prognostic Score; CEA, Carcinoembryonic antigen; CA19-9 carbohydrate antigen 19-9; SIR, systemic inflammatory response; STM score, Stroma-Tumor Marker Score.

As for local relapse free survival, CEA was the only variable that stratified survival significantly (*p* = 0.009), and also a tendency for high PLR (*p* = 0.087) was observed (Supplementary Table S6). For distant metastasis free survival, TSR (*p* = 0.017) and serum albumin (*p* = 0.031) were associated with survival, while there was a tendency towards poor survival with GMS (*p* = 0.057), CEA (*p* = 0.066) and CRP (*p* = 0.092). Figure 6.

In the univariate Cox regression analysis TSR, GMS, mGPS, ANC, CRP, Albumin, CEA and CA19-9 were significantly associated with OS; CMS presented a tendency (with a *p* = 0.055, just failing to be significant) towards increased risk of death in CMS4 patients and NLR and NPS also showed tendency for poorer OS (Supplementary Table S7).

In the multivariate analysis, TSR (*p* = 0.029), NPS (*p* = 0.033), mGPS (*p* = 0.003), Albumin (*p* = 0.003), CRP (*p* = 0.018), CA19-9 (*p* = 0.013), and STM-score (*p* ≤ 0.001) were significant predictors of OS (independently of sex, grade, stage and vascular invasion) (Supplementary Table S7).

### STM score

Scoring systems comprising of semi-quantitative aspects of certain TME or systemic inflammation-based markers are no novelty in oncology. A combination of strong prognostic factors have the ability to further identify a subset of patients with particularly poor outcome.

In our research the strongest independent TME-based marker was the TSR (Supplementary Table S7). Also, CA19-9, a tumor marker, often, though not routinely used in colorectal cancer follow up, came out as a predictor of overall survival in our analysis (Supplementary Table S7). Incorporating these two, STM was created. The assessment of STM is described in Methods previously.

Cases classified as STM2 were associated with younger age (*p* = 0.014), higher pN (*p* = 0.033) and M (*p* < 0.001), as well as higher TNM stage (*p* < 0.001), and presence of lymphatic (*p* < 0.001) and perineural invasion (*p* < 0.001), and also with elevated CEA-levels (*p* = 0.002) (Table 4). The mesenchymal subtype of CMS was also more prevalent in STM1 and STM2 groups (*p* = 0.048) (Table 4). There was a tendency towards higher pT (*p* = 0.071), also, preoperative serum CRP and CEA levels correlated with STM1 and STM2 (*p* = 0.017 and *p* < 0.001) (Table 4 and Supplementary Table S8).

**TABLE 4:** The relationship between stroma-tumor marker (STM) score and clinicopathological features.

Clinico-pathological features	All patients (n = 135)	STM score (n = 135)			
		STM 0 (n = 80)	STM 1 (n = 43)	STM 2 (n = 12)	*p*-value
Age					**0.014**
<65	46 (34%)	21 (26%)	16 (37%)	9 (75%)	
65-74	60 (44%)	42 (53%)	16 (37%)	2 (17%)	
75<	29 (22%)	17 (21%)	11 (26%)	1 (8%)	
Sex					0.877
Female	73 (54%)	44 (55%)	22 (51%)	7 (58%)	
Male	62 (46%)	36 (45%)	21 (49%)	5 (42%)	
Location					0.288
Right colon	57 (42%)	36 (45%)	16 (37%)	5 (42%)	
Left colon	44 (33%)	26 (33%)	12 (28%)	6 (50%)	
Rectum	34 (25%)	18 (23%)	15 (35%)	1 (8%)	
pT					*0.071*
pT1	2 (1.5%)	2 (3%)	0 (0%)	0 (0%)	
pT2	25 (19%)	20 (25%)	5 (12%)	0 (0%)	
pT3	97 (72%)	55 (69%)	32 (74%)	10 (83%)	
pT4	11 (8%)	3 (4%)	6 (14%)	2 (17%)	
pN					**0.033**
pN0	58 (43%)	41 (52%)	16 (37%)	1 (8%)	
pN1	51 (38%)	27 (34%)	18 (42%)	6 (50%)	
pN2	25 (19%)	11 (14%)	9 (21%)	5 (42%)	
M					**<0.001**
M0	104 (77%)	73 (91%)	28 (65%)	3 (25%)	
M1	31 (23%)	7 (9%)	15 (35%)	9 (75%)	
Stage					**<0.001**
I	21 (16%)	17 (23%)	4 (9%)	0 (0%)	
II	30 (22%)	22 (28%)	8 (19%)	0 (0%)	
III	53 (39%)	34 (43%)	16 (37%)	3 (25%)	
IV	31 (23%)	7 (9%)	15 (35%)	9 (75%)	
Grade					0.916
Low/moderate	119 (88%)	70 (88%)	38 (88%)	11 (92%)	
High	19 (12%)	10 (13%)	5 (12%)	1 (8%)	
Lymphatic invasion					**<0.001**
Not present	89 (64%)	62 (78%)	20 (47%)	4 (33%)	
Present	49 (36%)	18 (23%)	23 (54%)	8 (67%)	
Perineural invasion					**0.001**
Not present	121 (90%)	78 (98%)	34 (79%)	9 (75%)	
Present	14 (10%)	2 (3%)	9 (21%)	3 (25%)	
Vascular invasion					0.440
Not present	103 (76%)	64 (80%)	31 (72%)	8 (67%)	
Present	32 (24%)	16 (20%)	12 (28%)	4 (33%)	
CMS dMMR	12 (11%)	10 (15%)	2 (6%)	0 (0%)	**0.048**
Epithelial	75 (69%)	48 (73%)	20 (59%)	7 (78%)	
Mesenchymal	22 (20%)	8 (12%)	12 (25%)	2 (22%)	
mGPS					0.414
mGPS 0	37 (50%)	24 (60%)	9 (39%)	4 (36%)	
mGPS 1	26 (35%)	12 (30%)	9 (39%)	5 (46%)	
mGPS 2	11 (15%)	4 (10%)	5 (22%)	2 (18%)	
CEA (n = 152)					**0.002**
CEA-low	90 (67%)	62 (78%)	24 (56%)	4 (33%)	
CEA-high	45 (33%)	18 (23%)	19 (44%)	8 (67%)	

The relationship between stroma-tumor marker (STM) score and clinicopathological features was assessed using Chi-squared test. Significant correlations were marked with bold font, while tendencies where *p* < 0.1 were marked with italic font. In some cases, percentages do not add up to 100% precisely due to rounding. Abbreviations: STM, stroma-tumor marker score; CMS, consensus molecular subtype; dMMR, mismatch repair deficient; mGPS, modified Glasgow prognostic score; CEA, carcinoembryonic antigen.

The STM score significantly stratified 5-year overall survival (86% versus 54% versus 42%) with Kaplan-Meier analysis (Supplementary Table S6). Also in stage I-III patients there was significant difference between the distant metastasis free survival of STM0, 1 and 2 patients (*p* = 0.005) (Supplementary Table S6). In the univariate Cox-regression analysis STM was significantly associated with OS (HR: 7.4 (3-18), *p* < 0.001), and in the multivariate Cox-regression analysis STM was found to be an independent prognosticator of OS independently of sex, grade, stage and vascular invasion (*p* < 0.001, HR: 4.3 (1.5-12) (Supplementary Table S7).

## Discussion

In our study we characterized TME with TSR, KM-grade and their combination, the GMS grading system, which are all convenient descriptors to use and present good reproducibility. Similarly to previous studies [10, 12, 34], stroma-high tumors represented an aggressive phenotype with poor prognosis and inferior survival in this cohort. According to literature data, higher KM grade is associated with favorable clinicopathological features [6, 34], which was the case in our study as well, though a significant association with OS could not be presented. Although KM grade was described to be related to systemic inflammation [35], this finding was not observed in our study. Similarly to TSR, GMS also successfully stratified patients’ characteristics and survival, in agreement with preceding results [36]. In conclusion, the aformentioned and easily assessible descriptors, TSR, KM-grade and GMS, can guide us in CRC-prognostication.

The pre-operative systemic inflammation can be described using a variety of SIR markers. As described in previous reports [23, 37], some of the SIR markers were associated with poor patient outcomes in our cohort as well. Four of them (CRP, albumin, mGPS and NPS) even came out as independent factors of overall survival. Amongst these markers CRP not only delivered robust prognostic power, but was also associated with adverse histological features and advanced stages. These results suggest that CRP represented the effect of inflammatory response on clinical outcome the most, which is not surprising, as CRP is a key acute phase protein of inflammatory processes [38].

Another substantial finding regarding SIR markers was that APC elevation correlated with male sex and right sidedness. Interestingly, women generally tend to have higher APC than men [39]. It is also understood that higher APC is associated with poorer survival in CRC patients [40]. In our study, men also had worse OS, than women (*p* = 0.039 using log-rank test, mean OS (men): 6.3 years, OS (women): 8.3 years). We believe, elevated APC possibly indicated the poorer outcome of men in our cohort. Another observation that might explain our findings is that APC is significantly higher in dMMR CRC than in proficient MMR tumors [35], and dMMR CRCs are associated with right sidedness [35].

Tumor markers CEA and CA19-9 and their relationship with TME and SIR was also assessed. In concordance with previous articles [41, 42], our study also showed that both CEA and CA19-9 were linked to advanced stages of CRC and CA19-9 even emerged as an independent factor of OS.

Surprisingly, both CEA and CA19-9 showed a statistically significant association with TSR, but not with KM grade and CMS, which were not yet reported elsewhere. The connection between TSR-high tumors and elevated tumor markers could be attributed to the higher presence of distant metastasis or locally advanced disease indicated by both markers.

Important to point out, that both TSR and CA19-9 delivered strong prognostic value, whichproposed a possible combined score, the STM, that bears similar or even stronger prognostic power than these two variables separately. STM was strongly associated with dismal clinicopathological parameters and proved to be the second best prognosticator of OS. In conclusion, combined scores based on histopathological features and routine laboratory tests, like mGPS or STM, could help to identify a subset of CRC patients with higher risk of death or recurrence in a cost-effective and time sparing manner.

Reportedly, our study is the first one to assess the connection between CMS and SIR markers. CMS1 displays a characteristic inflammatory infiltrate that could lead to systemic inflammation which may be reflected in elevated SIR markers [35]. In our analysis, CMS1 was associated with right-sidedness and elevated APC, similarly to previous findings [21], and it correlated with NPS and PLR as well.

It is understood that CMS4 is associated with EMT-like gene expression profile and stromal infiltration signature [14], complement components and immunosuppressive chemokines [15]. This signature is similar to wound healing responses or chronic tumor-supportive inflammation, where platelets are the first responders and several mediators present in CMS4 tumors are associated with to platelet activation [43], hence an elevated APC was expected amongst mesenchymal CRCs. Interestingly, CMS4 tumors did not exhibit elevation of any platelet-related, nor any other SIR markers in our cohort. In conclusion, no significant association between CMS and inflammation was found. A recent paper emphasizes the diversity of immunological subtypes and their distribution within CMS, that might provide an explanation as to why there is a lack of distinct SIR-related characteristic of each molecular subtype [44]. Surprisingly, this research associates CMS2 (also, most epithelial-like CRCs) with a dominant wound healing-like immune response, while in CMS4 tumors such an immunological profile is less frequent [44], which is contradictory with the previously mentioned report [43]. Apparently, the rationale and exact mechanisms behind the resemblance of SIR-profiles of CMS-classified tumors are to be further examined.

Also, our study assigned poorer survival and higher TNM-stages to CMS4-tumors, which is similar to literature data showing that advanced CRCs are enriched in CMS4 [14, 45]. A possible pitfall of CMS classification could be the intratumoral heterogeneity and EMT, especially if samples derive from the invasive front of the tumor, which can lead to misclassifying cases as CMS4 [46]. To avoid sampling bias, our TMA cores were selected from the tumor centre. As of now, it is still difficult to answer whether CMS4 is the cause or the consequence of advanced CRCs. A study on exploring the relationship between interval CRC and CMS or more precise studies dealing with heterogeneity within tumor areas (e.g.,: multiple sampling from more tumor areas) could answer these questions.

No significant association between traditional tumor markers and CMS was found in our research. Another article found that in stage III CMS4 CRCs, elevated CEA was associated with exceptionally poor prognosis, and suppressed tumor immunity was also observed in this subgroup [47]. Similar analysis could not be performed in our database, as there were only 11 stage III CMS4 cases.

A probable limitation of our study was the relatively small cohort size sometimes resulting few cases in the subclasses (especially for CMS4) and weak to moderate statistical power, as well as the retrospective nature of inclusion of stage IV patients. In addition, the immunohistochemistry-based approach was used for CMS classification, which is simple and cost-effective, presenting 87% of concordance with the gold-standard gene-expression based profiling, and CMS2 and CMS3 cannot be distingiushed [32, 33].

## Conclusion

In conclusion, our results are in line with the literature data claiming that most TME, SIR markers and elevated CEA or CA19-9 are associated with adverse histological features and patient outcome. The authors’ work further broadens the potential options of cost-effective, evidence based prognostic tools. Assessing and combining routine histopathology (TSR) with laboratory findings (CA19-9) resulted in a novel, robust prognostic score, the STM score, which could be a simple and easily accessible risk stratificator. The authors believe this could be useful in identifying subsets of CRC patients who benefit from more intensive therapy to prevent recurrence or progression.

As in previous studies, CMS4 tumors represented an aggressive phenotype of CRC with adverse histological features and poor patient outcome, which was also reflected by its association with higher TSR. This further confirms the versatility of TSR assessment and its potential role in identifying patients at risk or cases with high probability of CMS4.

Up to now only very few studies investigated the connection between CMS and TME, SIR and tumor markers. Contrary to the authors’ expectations, CMS4 and CMS2/3 were not associated with any SIR nor tumor markers, only dMMR (CMS1) tumors correlated with plateled derived SIR markers, as described previously. This underlines the complexity of tumor-host response and proposes possible future investigations of this field.

## Data Availability

The raw data supporting the conclusion of this article will be made available by the authors, without undue reservation.

## Footnotes

1
https://crcclassifier.shinyapps.io/appTesting/

## Abbreviations

ALC, absolute lymphocyte count; ANC, absolute neutrophil count; APC, absolute platelet count; CA19-9, Carbohydrate antigen; CEA, Carcinoembryonic antigen; CK, Cytokeratin; CMS, Consensus molecular subtype; CRC, colorectal cancer; CRP, C reactive protein; dMMR, mismatch repair deficient; EMT, Epithelial-mesenchymal transition; FFPE, formalin fixed paraffin embedded; GMS, Glasgow microenvironment score; HE, hematoxylin-eosin; KM score, Klintrup-Makinen score; M, distant metastasis; mGPS, modified Glasgow prognostic score; MSI, microsatellite instability or instable; NLR, neutrophil—lymphocyte ratio; NPS, Neutrophil—platelet score; OS, overall survival; PLR, platelet—lymphocyte ratio; pMMR, proficient MMR; pN, pathological, regional lymph node metastasis; pT, pathological, extent of primary tumor; SIR, systemic inflammatory response; STM, Stroma-tumor marker; TMA, Tissue Microarray; TME, Tumor microenvironment; TSR, Tumor-stroma ratio.
