# Supplementary material for: Microenvironment, systemic inflammatory response and tumor markers considering consensus molecular subtypes of colorectal cancer
Source: Pathol Oncol Res. 2024 Apr 5;30:1611574. doi: 10.3389/pore.2024.1611574 (PMC11026638; doi:10.3389/pore.2024.1611574)
Supplement: Supplementary file 1 [file DataSheet4.DOCX]

Supplementary table 4: The relationship between clinicopathological features, TME markers and composite ratios and cumulative scores (mGPS, NLR, PLR, NPS)

| Clinico-pathological features | mGPS (n=95) | | | | NLR (n=170) | | | PLR (n=168) | | | NPS (n=168) | | | |
| --- | --- | --- | --- | --- | --- | --- | --- | --- | --- | --- | --- | --- | --- | --- |
|  | mGPS0  (n=41) | mGPS1  (n=34) | mGPS2  (n=20) | p value | NLR-low  (n=88) | NLR-high  (n=82) | p value | PLR-low  (n=92) | PLR-high  (n=76) | p value | NPS0  (n=85) | NPS1  (n=69) | NPS2  (n=24) | p value |
| Age (n=185)  <65  65-74  75< | 10 (24%)  25 (61%)  6 (15%) | 12 (35%)  11 (32%)  11 (32% | 8 (40%)  7 (25%)  5 (35% | p=0.100 | 34 (39%)  34 (39%)  20 (23%) | 23 (28%)  40 (49%)  19 (23%) | p=0.297 | *36 (39%)*  *33 (36%)*  *23 (25%)* | *20 (26%)*  *40 (53%)*  *16 (21%)* | *p=0.081* | 27 (36%)  27 (36%)  21 (28%) | 22 (32%)  35 (51%)  12 (17%) | 7 (29%)  11 (46%)  6 (25%) | p=0.414 |
| Sex (n=185)  Female  Male | 20 (49%)  21 (50%) | 21 (62%)  13 (38%) | 13 (65%)  7 (35%) | p=0.374 | 47 (53%)  41 (47%) | 42 (51%)  40 (49%) | p=0.775 | 49 (53%)  43 (47%) | 39 (51%)  37 (49%) | p=0.802 | **41 (55%)**  **34 (45%)** | **42 (61%)**  **27 (39%)** | **5 (21%)**  **19 (79%)** | **p=0.003** |
| Location  (n=185)  Right colon  Left colon  Rectum | 14 (34%)  13 (32%)  14 (34%) | 14 (41%)  9 (27%)  11 (32%) | 8 (40%)  9 (45%)  3 (15%) | p=0.488 | 35 (40%)  28 (32%)  25 (28%) | 34 (42%)  27 (33%)  21 (26%) | p=0.919 | *31 (34%)*  *31(34%)*  *30 (33%)* | *38 (50%)*  *24 (32%)*  *14 (18%)* | *p=0.051* | 29 (39%)  24 (32%)  22 (29%) | 27 (39%)  23 (33%)  19 (28%) | 13 (54%)  8 (33%)  3 (13%) | p=0.518 |
| pT (n=185)  pT1  pT2  pT3  pT4 | *1 (2%)*  *10 (24%)*  *29 (71%)*  *1 (2%)* | *1 (3%)*  *5 (15%)*  *21 (62%)*  *7 (21%)* | *0 (0%)*  *0 (0%)*  *16 (80%)*  *4 (20%)* | *p=0.057* | 2 (2%)  19 (22%)  63 (72%)  4 (5%) | 0 (0%)  13 (16%)  60 (73%)  9 (11%) | p=0.178 | 1 (1%)  16 (17%)  69 (75%)  6 (7%) | 1 (1%)  15 (20%)  53 (70%)  7 (9%) | p=0.876 | **2 (3%)**  **18 (24%)**  **53 (71%)**  **2 (3%)** | **0 (0%)**  **10 (15%)**  **53 (77%)**  **6 (9%)** | **0 (0%)**  **3 (19%)**  **16 (73%)**  **5 (8%)** | **p=0.043** |
| pN (n=185)  pN0  pN1  pN2 | 18 (44%)  18 (44%)  5 (12%) | 13 (38%)  15 (44%)  6 (18%) | 8 (40%)  7 (35%)  5 (25%) | p=0.775 | 37 (43%)  30 (35%)  20 (23%) | 35 (43%)  34 (42%)  13 (16%) | p=0.440 | 35 (38%)  38 (41%)  19 (21%) | 36 (48%)  26 (35%)  13 (17%) | p=0.443 | **38 (51%)**  **20 (27%)**  **16 (22%)** | **21 (30%)**  **36 (52%)**  **12 (17%)** | **12 (50%)**  **8 (33%)**  **4 (17%)** | **p=0.032** |
| M (n=185)  M0  M1 | 31 (76%)  10 (24%) | 25 (74%)  9 (27%) | 12 (60%)  8 (40%) | p=0.425 | 69 (78%)  19 (22%) | 62 (76%)  20 (24%) | p=0.664 | 74 (80%)  18 (20%) | 56 (77%)  20 (23%) | p=0.298 | 60 (80%)  15 (20%) | 55 (80%)  14 (20%) | 15 (63%)  9 (38%) | p=0.170 |
| Stage (n=185)  I  II  III  IV | 7 (17%)  8 (20%)  16 (39%)  10 (24%) | 3 (9%)  9 (27%)  13 (38%)  9 (27%) | 0 (0%)  6 (30%)  6 (30%)  8 (40%) | p=0.408 | 15 (17%)  22 (25%)  32 (36%)  19 (22%) | 10 (12%)  19 (23%)  33 (30%)  20 (24%) | p=0.789 | 12 (13%)  21 (23%)  41 (45%)  18 (20%) | 12 (16%)  20 (26%)  24 (32%)  20 (26%) | p=0.379 | *15 (20%)*  *22 (29%)*  *23 (31%)*  *15 (20%)* | *6 (9%)*  *14 (20%)*  *35 (51%)*  *4 (20%)* | *3 (13%)*  *5 (21%)*  *7 (29%)*  *9 (38%)* | *p=0.067* |
| Grade (n=185)  Low/mode-rate  High | **36 (88%)**  **5 (12%)** | **32 (94%)**  **2 (6%)** | **14 (70%)**  **6 (30%)** | **p=0.042** | 78 (89%)  10 (11%) | 72 (88%)  10 (12%) | p=0.866 | 83 (90%)  9 (10%) | 65 (86%)  11 (15%) | p=0.350 | 67 (89%)  8 (11%) | 59 (86%)  10 (15%) | 22 (92%)  2 (8%) | p=0.656 |
| Lymphatic invasion (n=185)  Not present  Present | 31 (76%)  10 (24% | 18 (53%)  16 (47%) | 12 (60%)  8 (40%) | p=0.114 | 59 (67%)  29 (33%) | 53 (65%)  29 (35%) | p=0.740 | 63 (69%)  29 (32%) | 47 (62%)  29 (38%) | p=0.368 | 53 (71%)  22 (29%) | 41 (59%) 28 (41%) | 16 (67%)  8 (33%) | p=0.363 |
| Perineural invasion (n=185)  Not present  Present | 37 (90%)  4 (10%) | 31 (91%)  3 (9%) | 17 (85%)  3 (15%) | p=0.757 | 82 (93%)  6 (7%) | 74 (90%)  8 (10%) | p=0.486 | 86 (94%)  6 (7%) | 68 (90%)  8 (11%) | p=0.350 | 70 (93%)  5 (7%) | 63 (91%)  6 (9%) | 21 (88%)  3 (13%) | p=0.660 |
| Vascular invasion (n=185)  Not present  Present | 35 (85%)  6 (15%) | 24 (71%)  10 (29%) | 14 (70%)  6 (30%) | p=0.229 | 66 (75%)  22 (25%) | 65 (79%)  17 (20%) | p=0.508 | 71 (77%)  21 (23%) | 59 (77%)  17 (22%) | p=0.944 | 60 (80%)  15 (20%) | 52 (77%)  16 (23%) | 17 (71%)  7 (29%) | p=0.639 |
| CMS (n=73)  dMMR  Epithelial  Mesenchymal | 2 (6%)  24 (67%)  10 (28%) | 3 (12%)  17 (68%)  5 (20%) | 0 (0%)  14 (82%)  3 (18%) | p=0.486 | 6 (8%)  54 (74%)  13 (18%) | 9 (13%)  45 (66%)  14 (21%) | p=0.527 | 4 (6%)  54 (74%)  15 (21%) | 11 (16%)  44 (66%)  12 (18%) | p=0.112 | **3 (5%)**  **47 (77%)**  **11 (18%)** | **6 (10%)**  **41 (71%)**  **11 (19%)** | **6 (29%)**  **10 (48%)**  **5 (24%)** | **p=0.034** |
| TSR (n=185)  TSR-low  TSR-high | 29 (71%)  12 (29%) | 20 (59%)  14 (41%) | 11 (55%)  9 (45%) | p=0.395 | 53 (71%)  22 (29%) | 41 (59%)  28 (41%) | p=0.273 | 56 (61%)  36 (39%) | 51 (67%)  25 (33%) | p=0.403 | 50 (67%)  25 (33%) | 41 (59%)  28 (41%) | 16 (67%)  8 (33%) | p=0.630 |
| KM grade (n=185)  KM-low  KM-high | 27 (66%)  14 (34%) | 25 (74%)  9 (27%) | 15 (75%)  5 (25%) | p=0.680 | 59 (67%)  29 (33%) | 56 (68%)  26 (32%) | p=0.862 | 63 (69%)  29 (32%) | 51 (67%)  25 (33%) | p=0.850 | 51 (68%)  24 (32%) | 46 (67%)  23 (33%) | 17 (71%)  7 (29%) | p=0.931 |
| GMS  (n=185)  GMS 0  GMS 1  GMS 2 | 25 (61%)  7 (17%)  9 (22%) | 16 (47%)  7 (21%)  11 (32%) | 7 (35%)  7 (35%)  6 (30%) | p=0.316 | 48 (55%)  17 (19%)  23 (26%) | 45 (55%)  20 (24%)  17 (21%) | p=0.598 | 48 (52%)  19 (21%)  25 (27%) | 43 (57%)  18 (24%)  15 (20%) | p=0.525 | 41 (55%)  19 (25%) 15 (20%) | 37 (54%)  15 (22%)  17 (25%) | 13 (54%)  3 (13%)  8 (33%) | p=0.598 |

The relationship between certain clinicopathological features (including CMS, TSR, KM grade, GMS) and composite ratios and cumulative score was examined using Chi-squared test. Significant correlations were marked with bold font, tendencies where p<0.1 were marked with italic font.

Abbreviations: mGPS – modified Glasgow prognostic score, NLR – neutrophil-lymphocyte ratio, PLR – platelet-lymphocyte ratio, NPS – neutrophil-platelet score, CMS – consensus molecular subtype, TSR – tumor-stroma ratio, KM – Klintrup-Makinen grade, GMS – Glasgow microenvironment score
