# Supplementary material for: Microenvironment, systemic inflammatory response and tumor markers considering consensus molecular subtypes of colorectal cancer
Source: Pathol Oncol Res. 2024 Apr 5;30:1611574. doi: 10.3389/pore.2024.1611574 (PMC11026638; doi:10.3389/pore.2024.1611574)
Supplement: Supplementary file 2 [file DataSheet7.DOCX]

Supplementary table 7: Results of uni- and multivariate Cox regression analysis of examined TME, SIR and tumor markers regarding overall survival

|  | Univariate analysis for OS HR (95% CI) | p-value | Variables included in multivariate analysis | Multivariate analysis for OS HR (95% CI)^*^ | p-value |
| --- | --- | --- | --- | --- | --- |
| TSR (low/high) | **2.7 (1.6-44)** | **<0.001** | **TSR**  Sex  Grade  Vascular invasion  **Stage** | **1.8 (1.1-3)**  0.7 (0.3-1.5)  1.8 (1-3.5)  1.7 (0.8-3.9)  **5.6 (1.6-20)** | **0.029** |
| KM grade (low/high) | 1.1 (0.6-1.8) | 0.763 | - | - | - |
| GMS (0/1/2) | **2.3 (1.3-4)** | **0.004** | GMS  Sex  Grade  Vascular invasion  Stage | 1.4 (0.8-2.6)  0.6 (0.4-1)  1.9 (1-3.7)  1.9 (1-3.3)  6.7 (1.9-23) | 0.130 |
| CMS (dMMR/Epithelial/Mesenchymal) | *2.2 (0.6-6.8)* | *0.055* | CMS  Sex  Grade  Vascular invasion  Stage | 1.7 (0.5-5.4)  0.6 (0.4-1)  1.9 (1-4)  1.8 (1-3.4)  4.7 (1.4-17) | 0.371 |
| ANC (low/high) | **2.1 (1.3-3.6)** | **0.007** | *ANC*  Sex  Grade  **Vascular invasion**  **Stage** | *1.7 (0.99-3)*  0.6 (0.3-1)  1.4 (0.7-2.8)  **2 (1.1-3.5)**  **2.4 (1.7-3.5)** | *0.055* |
| ALC (low/high) | 0.8 (0.5-1.4) | 0.445 | - | - | - |
| APC (low/high) | 1.3 (0.7-2.4) | 0.399 | - | - | - |
| NLR (low/high) | *1.6 (0.9-2.7)* | *0.085* | NLR  Sex  Grade  Vascular invasion  Stage | 1.4 (0.2-2.4)  0.6 (0.4-1)  1.3 (0.6-2.7)  1.9 (1.1-3.5)  7.7 (2.3-26) | 0.194 |
| PLR (low/high) | 1.4 (0.8-2.4) | 0.209 | - | - | - |
| NPS (low/high) | *2 (0.9-4.3)* | *0.072* | **NPS**  **Sex**  Grade  **Vascular invasion**  **Stage** | **1.6 (1-2.4)**  **0.5 (0.3-0.9)**  1.5 (0.7-3)  **2 (1.2-3.7)**  **6.8 (2-23)** | **0.033** |
| mGPS (0/1/2) | **4.8 (1.9-12)** | **0.004** | **mGPS**  Sex  Grade  Vascular invasion  **Stage** | **5.6 (2.1-15)**  0.7 (0.3-1.5)  1.3 (0.5-3.5)  1.7 (0.7-4)  **5 (1.9-21)** | **0.003** |
| Albumin (low/high) | **0.5 (0.3-0.9)** | **0.030** | **Albumin**  Sex  Grade  Vascular invasion  Stage | **0.5 (0.2-0.9)**  0.7 (0.3-1.4)  1.4 (0.6-3.3)  1.8 (0.9-3.9)  **3.9 (1.7-18)** | **0.022** |
| CRP (low/high) | **2 (1.2-3.5)** | **0.008** | **CRP**  Sex  Grade  **Vascular invasion**  **Stage** | **1.9 (1.1-3.4)**  0.7 (0.4-1.1)  1.9 (1-4)  **1.9 (1.1-3.5)**  **5.5 (1.2-24)** | **0.018** |
| CEA (low/high) | **2.9 (1.7-5.2)** | **<0.001** | *CEA*  Sex  Grade  **Vascular invasion**  **Stage** | *1.7 (0.9-3.3)*  0.6 (0.3-1.1)  **1.9 (1.1-3.6)**  **6.6 (1.4-30)** | *0.087* |
| CA 19-9 (low/high) | **3.9 (2-7.5)** | **<0.001** | **CA19-9**  **Sex**  **Grade**  **Vascular invasion**  **Stage** | **2.8 (1.2-6.1)**  0.5 (0.3-0.9)  1.7 (0.7-4)  **1.9 (1.1-3.8)**  **10 (1.3-81)** | **0.013** |
| Stroma-Tumor Marker score (0/1/2) | **7.4 (3-18)** | **<0.001** | **STM score**  **Sex**  Grade  **Vascular invasion**  **Stage** | **4.3 (1.6-11.9)**  **0.3-0.9**  1.7 (0.7-4)  **2 (1-3.8)**  **2.7 (1.2-6.1)** | **<0.001** |

Significant variables were marked with bold font, tendencies with p<0.1 were marked with italic font.

*: all variables were assessed individually in a multivariate analysis including sex, stage, grade and vascular invasion using enter method.

Abbreviations: TSR – tumor-stroma ratio, KM grade – Klintrup-Makinen grade, GMS – Glasgow microenvironment score, CMS – consensus molecular subtype, dMMR – mismatch repair deficient, ANC – absolute neutrophil count, ALC – absolute lymphocyte count, NLR – neutrohpil-lymphocyte ratio, PLR – platelet-lymphocyte ratio, NPS – neutrophil-platelet score, mGPS – modified Glasgow prognostic score, CRP – C reactive protein, CEA – carcinoembryonic antigen, CA 19-9 – carbohydrate antigen 19-9, STM – Stroma-Tumor Marker score

Abbreviations: TSR – tumor-stroma ratio, KM grade – Klintrup-Makinen grade, GMS – Glasgow microenvironment score, CMS – consensus molecular subtype, dMMR – mismatch repair deficient, ANC – absolute neutrophil count, ALC – absolute lymphocyte count, NLR – neutrohpil-lymphocyte ratio, PLR – platelet-lymphocyte ratio, NPS – neutrophil-platelet score, mGPS – modified Glasgow prognostic score, CRP – C reactive protein, CEA – carcinoembryonic antigen, CA 19-9 – carbohydrate antigen 19-9, STM – Stroma-Tumor Marker score
