# Supplementary material for: Microenvironment, systemic inflammatory response and tumor markers considering consensus molecular subtypes of colorectal cancer
Source: Pathol Oncol Res. 2024 Apr 5;30:1611574. doi: 10.3389/pore.2024.1611574 (PMC11026638; doi:10.3389/pore.2024.1611574)
Supplement: Supplementary file 3 [file DataSheet6.DOCX]

Supplementary table 6: The relationship between TME, SIR markers, CMS and survival parameters

|  | 5 year OS, stage I-IV  (median survival, p value) | 5 year LRFS, stage I-III  (median survival, p value) | 5 years DMFS, stage I-III  (median survival, p value) |
| --- | --- | --- | --- |
| TSR (n=185) | **TSR-low: 76%**  **TSR-high: 46%**  **p<0.001** | TSR-low: 87%  TSR-high: 77%  p=0.344 | **TSR-low: 84%**  **TSR-high: 63%**  **p=0.017** |
| KM grade (n=185) | KM low: 67%  KM high: 62%  p=0.763 | KM low: 88%  KM high: 80%  p=0.177 | KM low: 82%  KM high: 74%  p=0.166 |
| GMS (n=185) | **GMS 0: 70%**  **GMS 1: 68%**  **GMS 2: 48%**  **p=0.003** | GMS 0: 87%  GMS 1: 84%  GMS 2: 77%  p=0.691 | *GMS 0: 84%*  *GMS 1: 74%*  *GMS 2: 58%*  *p=0.057* |
| CMS (n=155) | **dMMR: 73%**  **Epithelial: 72%**  **Mesenchymal: 50%**  **p=0.049** | dMMR: 92%  Epithelial: 87%  Mesenchymal: 72%  p=0.370 | dMMR: 86%  Epithelial: 80%  Mesenchymal: 82%  p=0.902 |
| CEA (n=155) | **CEA low: 80%**  **CEA high: 43%**  **p<0.001** | **CEA low: 92%**  **CEA high: 68%**  **p=0.009** | *CEA low: 82%*  *CEA high: 63%*  *p=0.066* |
| CA 19-9 (n=135) | **CA 19-9 low: 76%**  **CA 19-9 high: 0%**  **p<0.001** | CA 19-9 low: 86%  CA 19-9 high: 86%  p=0.860 | *CA 19-9 low: 80%*  *CA 19-9 high: 57%*  *p=0.095* |
| mGPS (n=95) | **mGPS 0: 83%**  **mGPS 1: 59%**  **mGPS 2: 38%**  **p=0.002** | mGPS 0: 93%  mGPS 1: 83%  mGPS 2: 83%  p=0.433 | mGPS 0: 83%  mGPS 1: 77%  mGPS 2: 61%  p=0.173 |
| CRP (n=149) | **CRP low: 73%**  **CRP high: 55%**  **p=0.007** | CRP low: 86%  CRP high: 80%  p=0.243 | *CRP low: 81%*  *CRP high: 66%*  *p=0.092* |
| Albumin (n=107) | **Albumin low: 51%**  **Albumin high: 73%**  **p=0.027** | Albumin low: 81%  Albumin high: 90%  p=0.259 | **Albumin low: 65%**  **Albumin high: 85%**  **p=0.031** |
| ANC (n=170) | **ANC low: 81%**  **ANC high: 56%**  **p=0.006** | ANC low: 89%  ANC high: 86%  p=0.448 | ANC low: 81%  ANC high: 75%  p=0.472 |
| ALC (n=170) | ALC low: 63%  ALC high: 70%  p=0.444 | ALC low: 92%  ALC high: 83%  p=0.116 | ALC low: 76%  ALC high: 80%  p=0.934 |
| APC (n=180) | APC low: 70%  APC high: 60%  p=0.398 | APC low: 87%  APC high: 81%  p=0.503 | APC low: 79%  APC high: 73%  p=0.697 |
| NLR (n=170) | *NLR low: 74%*  *NLR high: 62%*  *p=0.082* | NLR low: 84%  NLR high: 91%  p=0.284 | NLR low: 80%  NLR high: 76%  p=0.683 |
| PLR (n=168) | PLR low: 75%  PLR high: 61%  p=0.207 | *PLR low: 84%*  *PLR high: 94%*  *p=0.087* | PLR low: 76%  PLR high: 80%  p=0.663 |
| NPS (n=168) | *NPS 0: 81%*  *NPS 1: 59%*  *NPS 2: 57%*  *p=0.076* | NPS 0: 92%  NPS 1: 84%  NPS 2: 86%  p=0.271 | NPS 0: 80%  NPS 1: 78%  NPS 2: 68%  p=0.623 |
| Stroma-tumor marker score (n=135) | **STM0: 86%**  **STM1: 54%**  **STM2: 42%**  **p<0.001** | STM 0: 87%  STM 1: 64%  STM 2: 35%  p=0.314 | **STM 0: 87%**  **STM 1: 64%**  **STM 2: 35%**  **p=0.005** |

The effect of tissue microenvironment (TME) and systemic inflammation on overall, local relapse free and distant metastasis free survival was evaluated with Log-rank test. Significant variables were marked with bold font, tendencies where p<0.1 were marked with italic font.

Abbreviations: OS – overall survival, LRFS – local relapse free survival, DMFS – distant metastasis free survival, TSR – tumor-stroma ratio, KM grade – Klintrup-Makinen grade, GMS – Glasgow microenvironment score, CMS – consensus molecular subtype, ANC – absolute neutrophil count, ALC – absolute lymphocyte count, APC – absolute platelet count, NLR – neutrophil-lymphocyte ratio, PLR – platelet-lymphocyte ratio, NPS – neutrophil platelet sore, mGPS – modified Glasgow prognostic score, CRP – C reactive protein, CEA – carcinoembryonic antigen, CA 19-9 – carbohydrate antigen 19-9, STM – stroma- tumor marker score
