# Supplementary material for: Microenvironment, systemic inflammatory response and tumor markers considering consensus molecular subtypes of colorectal cancer
Source: Pathol Oncol Res. 2024 Apr 5;30:1611574. doi: 10.3389/pore.2024.1611574 (PMC11026638; doi:10.3389/pore.2024.1611574)
Supplement: Supplementary file 4 [file DataSheet3.DOCX]

Supplementary table 3: The relationship between clinicopathological features and quantitative systemic inflammation-related markers and canonical tumor markers

|  | Albumin  (n=107) | CRP  (n=149) | ANC  (n=170) | ALC  (n=170) | APC  (n=180) | NLR  (n=168) | PLR  (n=168) | CEA  (n=155) | CA19-9  (n=135) |
| --- | --- | --- | --- | --- | --- | --- | --- | --- | --- |
| Age  <65  65-74  75< | **p=0.039**  **(42)**  **(41.5)**  **(38.6)** | p=0.986  (12.2)  (4.3)  (7.7) | p=0.199  (4.87)  (4.84)  (4.54) | **p=0.007**  **(1.6)**  **(1.6)**  **(1.41)** | p=0.816  (263)  (250)  (242) | *p=0.056*  *(3.12)*  *(3.31)*  *(3.2)* | **p=0.046**  **(160)**  **(196)**  **(187)** | p=0.850  (8.8)  (2.1)  (3.3) | p=0.639  (118)  (17)  (11.4) |
| Sex  Female  Male | p=0.215  (41.2)  (40.9) | p=0.732  (6.1)  (7.7) | p=0.528  (4.7)  (4.9) | p=0.585  (1.63)  (1.54) | **p=0.003**  **(236)**  **(278)** | p=0.887  (2.97)  (3.2) | p=0.194  (181.5)  (193) | *p=0.099*  *(3.3)*  *(2.6)* | p=0.513  (13.6)  (20.5) |
| Location  Right colon  Left colon  Rectum | p=0.338  (39.8)  (41.8)  (41.1) | p=0.338  (9.7)  (3.3)  (5) | p=0.978  (4.8)  (4.8)  (4.7) | p=0.628  (1.6)  (1.7)  (1.3) | **p=0.013**  **(317)**  **(250)**  **(237)** | p=0.705  (3.2)  (3)  (3.2) | **p=0.022**  **(231)**  **(163)**  **(176)** | **p=0.033**  **(2.9)**  **(2.5)**  **(3.5)** | p=0.131  (14.4)  (26.1)  (17.2) |
| pT  pT1  pT2  pT3  pT4 | p=0.241  (40.1)  (42.6)  (40.4)  (41.4) | **p<0.001**  **(7.1)**  **(5.1)**  **(5.5)**  **(87.7)** | **p=0.011**  **(3.5)**  **(4.8)**  **(4.7)**  **(6.1)** | p=0.901  (1.4)  (1.7)  (1.5)  (1.7) | p=0.459  (270)  (278)  (244)  (293) | p=0.120  (2.5)  (3.2)  (3.2)  (3.8) | p=0.721  (195)  (180)  (182)  (288) | **p=0.044**  **(1.8)**  **(2.1)**  **(3.6)**  **(2.6)** | **p=0.002**  **(21)**  **(4.8)**  **(18)**  **(31)** |
| pN  pN0  pN1  pN2 | p=0.509  (41)  (41)  (40) | p=0.354  (4.5)  (9.1)  (9.8) | *p=0.058*  *(4.6)*  *(4.9)*  *(6.1)* | p=0.145  (1.3)  (1.7)  (1.7) | p=0.333  (250)  (237)  (308) | p=0.661  (3.6)  (3.1)  (2.7) | p=0.539  (196)  (182)  (152) | *p=0.062*  *(2.3)*  *(3.5)*  *(2.9)* | p=0.179  (18)  (14)  (20) |
| M  M0  M1 | p=0.895  (41)  (40) | **p=0.007**  **(5)**  **(13)** | p=0.457  (4.7)  (4.9) | p=0.800  (1.6)  (1.6) | *p=0.068*  *(250)*  *(274)* | p=0.508  (3.2)  (3) | p=0.442  (194)  (152) | **p<0.001**  **(2.2)**  **(9.5)** | **p<0.001**  **(11)**  **(78)** |
| Stage  I  II  III  IV | *p=0.089*  *(42)*  *(41)*  *(41)*  *(40)* | **p=0.002**  **(4.3)**  **(4)**  **(6)**  **(13)** | p=0.111  (4.7)  (4.6)  (5.2)  (4.9) | p=0.213  (1.5)  (1.2)  (1.8)  (1.6) | p=0.293  (278)  (231)  (263)  (274) | p=0.793  (3.4)  (3.5)  (3)  (3) | p=0.737  (180)  (212)  (167)  (152) | **p<0.001**  **(1.6)**  **(2.3)**  **(3.1)**  **(9.5)** | **p<0.001**  **(12)**  **(17)**  **(9.6)**  **(78)** |
| Grade  Low/mode-rate  High | p=0.579  (41)  (41) | **p=0.027**  **(6.1)**  **(7.7)** | p=0.288  (4.8)  (5.8) | p=0.906  (1.6)  (1.6) | p=0.220  (248)  (302) | p=0.494  (3.2)  (3.2) | p=0.333  (190)  (155) | p=0.877  (3.4)  (2.6) | p=0.812  (18)  (9.1) |
| Lymphatic invasion  Not present  Present | p=0.543  (41)  (40) | **p=0.032**  **(4.3)**  **(12)** | p=0.398  (4.6)  (6.1) | p=0.548  (1.5)  (1.6) | **p=0.045**  **(242)**  **(315)** | p=0.770  (3.2)  (3.2) | p=0.492  (177)  (250) | p=0.262  (2.9)  (3.4) | **p=0.015**  **(17)**  **(21)** |
| Perineural invasion  Not present  Present | p=0.992  (41)  (40) | *p=0.063*  *(5.2)*  *(12)* | p=0.847  (4.7)  (6.3) | p=0.708  (1.6)  (1.7) | p=0.228  (248)  (263) | p=0.883  (3.1)  (3.3) | p=0.774  (190)  (149) | p=0.198  (2.8)  (8.8) | *p=0.081*  *(17)*  *(140)* |
| Vascular invasion  Not present  Present | p=0.247  (41)  (39) | **p=0.026**  **(4.7)**  **(21)** | p=0.424  (4.6)  (6.3) | p=0.187  (1.6)  (1.5) | *p=0.077*  *(248)*  *(307)* | p=0.629  (3)  (3.5) | p=0.868  (190)  (232) | p=0.255  (2.5)  (3.3) | *p=0.065*  *(14)*  *(21)* |
| CMS (n=155)  dMMR  Epithelial  Mesenchymal | p=0.801  (43)  (41)  (40) | p=0.342  (11)  (4.7)  (8.6) | p=0.501  (5.9)  (4.6)  (5) | p=0.512  (1.6)  (1.3)  (1.7) | **p<0.001**  **(490)**  **(232)**  **(315)** | p=0.955  (3.7)  (3)  (2.6) | **p=0.005**  **(298)**  **(193)**  **(155)** | p=0.483  (2)  (2.9)  (3.4) | p=0.357  (12)  (18)  (11) |
| TSR  TSR-low  TSR-high | p=0.523  (41)  (41) | p=0.256  (4.3)  (9.1) | p=0.138  (4.6)  (6) | p=0.171  (1.4)  (1.7) | p=0.809  (250)  (272) | p=0.454  (3.2)  (2.9) | p=0.102  (195)  (155) | *p=0.062*  *(2.4)*  *(6)* | *p=0.072*  *(12)*  *(27)* |
| KM grade  KM-low  KM-high | p=0.606  (41)  (41) | p=0.386  (7.7)  (4.3) | p=0.206  (4.9)  (4.5) | p=0.939  (1.7)  (1.3) | p=0.524  (254)  (274) | p=0.415  (2.9)  (3.6) | p=0.943  (161)  (247) | p=0.906  (2.6)  (3.5) | p=0.782  (7.2)  (18) |
| GMS  GMS 0  GMS 1  GMS 2 | p=0.163  (42)  (38)  (40) | p=0.409  (4.3)  (2.8)  (19) | p=0.270  (4.6)  (4.7)  (6.2) | p=0.164  (1.6)  (1.3)  (1.8) | p=0.393  (274)  (227)  (291) | p=0.574  (3)  (3.2)  (3) | p=0.412  (196)  (195)  (152) | p=0.602  (2.6)  (2.2)  (4.9) | **p=0.027**  **(17)**  **(5.5)**  **(25)** |

The relationship between clinicopathological features and quantitative systemic inflammation-related markers and canonical tumor markers was examined using non-parametric tests (Mann-Whitney U-test and Kruskal-Wallis H-test). Significant relations were marked with bold font, tendencies where p<0.1 were marked with italic font. Numbers shown in parentheses denote median values of each continuous variable within the respective categorical variable group.

Abbreviations: CRP – C reactive protein, ANC – absolute neutrophil count, ALC – absolute lymphocyte count, APC, absolute platelet count, NLR – neutrophil-lymphocyte ratio, PLR – platelet-lymphocyte ratio, CEA – carcinoembryonic antigen, CA 19-9 – carbohydrate antigen 19-9, CMS – consensus molecular subtype, dMMR – mismatch repair deficient, TSR – tumor-stroma ratio, KM grade – Klintrup-Makinen grade, GMS – Glasgow microenvironment score.
