# Supplementary material for: Microenvironment, systemic inflammatory response and tumor markers considering consensus molecular subtypes of colorectal cancer
Source: Pathol Oncol Res. 2024 Apr 5;30:1611574. doi: 10.3389/pore.2024.1611574 (PMC11026638; doi:10.3389/pore.2024.1611574)
Supplement: Supplementary file 5 [file DataSheet5.DOCX]

Supplementary table 5: The relationship between clinicopathological features and CMS immunohistochemistry

|  | CK  (n=134) | | | FRMD6 (n=140) | | | CDX2  (n=138) | | | ZEB1  (n=139) | | | HTR2B  (n=138) | | | |
| --- | --- | --- | --- | --- | --- | --- | --- | --- | --- | --- | --- | --- | --- | --- | --- | --- |
|  | CK-low | CK-high | p-value | FRMD6-low | FRMD6-high | p-value | CDX2-low | CDX2-high | p-value | ZEB1 absent | ZEB1 present | p-value | HTR2B  low | HTR2B  intermed | HTR2B  high | p-value |
| Age  <65  65-74  75< | **30 (46%)**  **27 (41%)**  **6 (14%)** | **16 (24%)**  **32 (47%)**  **20 (29%)** | **p=0.012** | 16 (28%)  25 (43%)  17 (29%) | 31 (38%)  36 (44%)  15 (18%) | p=0.236 | 22 (34%)  29 (45%)  14 (20%) | 25 (34%)  32 (43%)  17 (23%) | p=0.928 | 40 (34%)  50 (42%)  28 (24%) | 7 (33%)  11 (52%)  3 (14%) | p=0.571 | *6 (32%)*  *4 (21%)*  *9 (47%)* | *27 (38%)*  *32 (44%)*  *13 (18%)* | *14 (30%)*  *24 (51%)*  *9 (19%)* | *p=0.050* |
| Sex  Female  Male | 37 (56%)  29 (44%) | 37 (54%)  31 (46%) | p=0.848 | 34 (59%)  24 (41%) | 43 (52%)  39 (48%) | p=0.469 | 32 (50%)  32 (50%) | 43 (58%)  31 (42%) | p=0.340 | 63 (53%)  55 (47%) | 13 (62%)  8 (38%) | p=0.470 | 10 (53%)  9 (47%) | 40 (56%)  32 (44%) | 25 (53%) 22 (47%) | p=0.956 |
| Location  Right colon  Left colon  Rectum | 18 (27%)  26 (39%)  22 (33%) | 29 (43%)  20 (29%)  19 (28%) | p=0.170 | 17 (29%)  21 (36%)  20 (35%) | 33 (40%)  27 (33%)  22 (27%) | p=0.3824 | 23 (36%)  17 (27%)  24 (38%) | 27 (37%)  29 (39%)  18 (24%) | p=0.165 | *43 (36%)*  *36 (31%)*  *39 (33%)* | *7 (33%)*  *11 (52%)*  *3 (14%)* | *p=0.098* | 6 (32%) 5 (26%)  8 (42%) | 24 (33%)  27 (38%)  21 (29%) | 20 (43%)  15 (32%)  12 (26%) | p=0.607 |
| pT  pT1  pT2  pT3  pT4 | 1 (2%)  9 (14%)  50 (76%)  6 (9%) | 1 (2%)  10 (15%)  52 (77%)  5 (7%) | p=0.985 | 1 (2%)  8 (14%)  43 (74%)  6 (10%) | 1 (1%)  14 (17%)  61 (74%)  6 (7%) | p=0.883 | *0 (0%)*  *8 (13%)*  *47 (73%)*  *9 (14%)* | *2 (3%)*  *14 (19%)*  *55 (74%)*  *3 (4%)* | *p=0.087* | *2 (2%)*  *15 (13%)*  *89 (75%)*  *12 (10%)* | *0 (0%)*  *7 (33%)*  *14 (67%)*  *0 (0%)* | *p=0.058* | 0 (0%)  3 (16%)  15 (79%)  1 (5%) | 1 (1%)  14 (19%)  51 (71%)  6 (8%) | 1 (2%)  5 (11%)  36 (77%)  5 (11%) | p=0.870 |
| pN  pN0  pN1  pN2 | 25 (39%)  26 (40%)  14 (22%) | 38 (56%)  19 (28%)  11 (16%) | p=0.131 | *31 (53%)*  *22 (38%)*  *5 (9%)* | *37 (46%)*  *24 (30%)*  *20 (25%)* | *p=0.050* | 29 (45%)  21 (33%)  14 (22%) | 39 (53%)  23 (32%)  11 (15%) | p=0.513 | 55 (47%)  42 (36%)  20 (17%) | 13 (62%)  3 (14%)  5 (24%) | p=0.150 | *12 (63%)*  *5 (26%)*  *2 (11%)* | *22 (47%)*  *29 (41%)*  *9 (13%)* | *23 (49%)*  *11 (23%)*  *13 (28%)* | *p=0.092* |
| M  M0  M1 | 43 (73%)  18 (27%) | 51 (75%)  17 (25%) | p=0.765 | 46 (79%)  12 (21%) | 58 (71%)  24 (29%) | p=0.253 | 45 (70%)  19 (30%) | 58 (78%)  16 (21%) | p=0.277 | 88 (75%)  30 (25%) | 16 (76%)  5 (24%) | p=0.875 | *18 (95%)*  *1 (5%)* | *54 (75%)*  *18 (25%)* | *32 (68%)*  *15 (32%)* | *p=0.075* |
| Stage  I  II  III  IV | 7 (11%)  18 (27%)  23 (35%)  18 (27%) | 8 (12%)  23 (34%)  20 (29%)  17 (25%) | p=0.829 | 6 (10%)  23 (40%)  17 (29%)  12 (21%) | 12 (15%)  20 (24%)  26 (32%)  24 (29%) | p=0.251 | 6 (9%)  21 (33%)  18 (28%)  19 (30%) | 12 (16%)  22 (30%)  24 (32%)  16 (22%) | p=0.489 | 13 (11%)  37 (31%)  38 (32%)  30 (25%) | 5 (24%)  6 (29%)  5 (24%)  5 (24%) | p=0.436 | 3 (16%)  9 (47%) 6 (32%)  1 (5%) | 11 (15%)  19 (26%)  24 (33%)  18 (25%) | 4 (9%)  15 (32%)  13 (28%)  15 (32%) | p=0.280 |
| Grade  Low/moderate  High | 58 (88%)  8 (12%) | 61 (90%)  8 (10%) | p=0.737 | 53 (91%)  5 (9%) | 72 (88%)  10 (12%) | p=0.501 | 56 (88%)  8 (13%) | 67 (91%)  7 (10%) | p=0.567 | 106 (90%)  12 (10%) | 18 (86%)  3 (14%) | p=0.575 | 17 (90%)  2 (11%) | 64 (89%)  8 (11%) | 42 (89%)  5 (11%) | p=0.995 |
| Lymphatic  invasion  Not present  Present | **38 (59%)**  **28 (41%)** | **52 (77%)**  **16 (24%)** | **p=0.020** | 41 (71%)  17 (29%) | 55 (67%)  27 (33%) | p=0.650 | **38 (59%)**  **26 (41%)** | **57 (77%)**  **17 (23%)** | **p=0.026** | 79 (67%)  39 (33%) | 16 (76%)  5 (24%) | p=0.402 | 15 (79%)  4 (21%) | 47 (65%)  25 (35%) | 32 (68%)  15 (32%) | p=0.524 |
| Perineural  invasion  Not present  Present | 58 (88%)  8 (12%) | 64 (94%)  4 (6%) | p=0.206 | 54 (93%)  4 (7%) | 74 (90%)  8 (10%) | p=0.650 | **55 (86%)**  **9 (14%)** | **71 (96%)**  **3 (4%)** | **p=0.037** | 108 (92%)  10 (9%) | 19 (91%)  2 (10%) | p=0.875 | 19 (100%)  0 (0%) | 63 (88%)  9 (13%) | 44 (94%)  3 (6%) | p=0.179 |
| Vascular  invasion  Not present  Present | 49 (74%)  17 (26%) | 53 (78%)  15 (22%) | p=0.616 | 48 (83%)  10 (17%) | 59 (72%)  23 (28%) | p=0.138 | 48 (75%)  16 (25%) | 57 (77%)  17 (23%) | p=0.781 | 91 (77%)  27 (23%) | 15 (71%)  6 (29%) | p=0.572 | 14 (74%)  5 (26%) | 55 (76%)  17 (24%) | 37 (79%)  10 (21%) | p=0.179 |
| KM grade  KM-low  KM-high | *49 (74%)*  *17 (26%)* | *41 (60%)*  *27 (40%)* | *p=0.086* | 38 (66%)  20 (35%) | 57 (70%)  25 (31%) | p=0.618 | 41 (64%)  23 (36%) | 53 (72%)  21 (28%) | p=0.342 | 79 (67%)  39 (33%) | 15 (71%)  4 (29%) | p=0.686 | 10 (53%)  9 (47%) | 53 (74%) 19 (26%) | 30 (64%)  17 (36%) | p=0.181 |
| GMS  GMS0  GMS1  GMS2 | 29 (44%)  16 (24%)  21 (32%) | 41 (60%)  15 (22%)  12 (17%) | p=0.105 | 34 (59%)  13 (22%)  11 (19% | 38 (46%)  22 (27%)  22 (27%) | p=0.341 | 36 (56%)  13 (20%)  15 (23%) | 36 (49%)  20 ((27%)  18 (24%) | p=0.595 | 60 (51%)  27 (22%)  31 (26%) | 13 (62%) 6 (29%) 2 (10%) | p=0.251 | 13 (68%)  3 (16%)  3 (16%) | 30 (42%) 22 (31%)  20 (28%) | 29 (62%)  8 (17%)  10 (21%) | p=0.126 |
| TSR  TSR-low  TSR-high | **33 (50%)**  **33 (50%)** | **51 (75%)**  **17 (25%)** | **p=0.003** | **43 (74%)**  **15 (26%)** | **47 (57%)**  **35 (43%)** | **p=0.041** | 40 (63%)  24 (38%) | 48 (65%)  26 (25%) | p=0.773 | 73 (62%)  45 (38%) | 16 (76%)  5 (24%) | p=0.208 | 15 (79%)  4 (21%) | 44 (61%)  28 (39%) | 29 (62%)  18 (38%) | p=0.333 |
| CEA  CEA-low  CEA-high | 39 (67%)  19 (33%) | 34 (64%)  19 (36%) | p=0.732 | **37 (80%)**  **9 (20%)** | **39 (57%)**  **30 (44%)** | **p=0.008** | *31 (59%)*  *22 (42%)* | *44 (73%)*  *16 (27%)* | *p=0.096* | 66 (70%)  29 (31%) | 10 (53%)  9 (47%) | p=0.155 | 8 (62%)  5 (39%) | 43 (69%)  19 (31%) | 25 (64%)  14 (36%) | p=0.790 |
| CA 19-9  CA 19-9-low  CA 19-9 high | 40 (83%)  6 (17%) | 36 (80%)  9 (20%) | p=0.678 | 34 (85%)  6 (15%) | 46 (81%)  11 (19%) | p=0.584 | 35 (78%)  10 (22%) | 43 (86%)  7 (14%) | p=0.297 | 64 (80%)  16 (20%) | 15 (94%)  1 (6%) | p=0.188 | 9 (90%)  1 (10%) | 45 (83%)  9 (17%) | 25 (78%)  7 (22%) | p=0.661 |
| CRP  CRP low  CRP high | 29 (60%)  19 (40%) | 36 (58%)  26 (42%) | p=0.803 | 25 (56%)  20 (44%) | 44 (63%)  26 (37%) | p=0.435 | 27 (53%)  24 (47%) | 41 (65%)  22 (35%) | p=0.189 | **54 (56%)**  **43 (44%)** | **14 (82%)**  **3 (18%)** | **p=0.039** | 11 (69%)  5 (31%) | 36 (60%)  24 (40%) | 20 (54%)  17 (46%) | p=0.599 |
| Albumin  Albumin low  Albumin high | 11 (29%)  27 (71%) | 14 (33%)  29 (67%) | p=0.726 | **16 (43%)**  **21 (57%)** | **10 (21%)**  **38 (79%)** | **p=0.026** | *9 (21%)*  *33 (79%)* | *16 (39%)*  *25 (61%)* | *p=0.081* | 23 (33%)  47 (67%) | 2 (15%)  11 (85%) | p=0.207 | 4 (40%)  6 (60%) | 17 (37%)  29 (63%) | 4 (15%)  23 (85%) | p=0.106 |
| GPS  GPS0  GPS1  GPS2 | 11 (38%)  12 (41%)  6 (21%) | 21 (50%)  10 (34%)  11 (26%) | p=0.289 | 12 (36%)  11 (33%)  10 (33%) | 23 (55%)  11 (26%)  8 (19%) | p=0.267 | 17 (47%)  11 (31%)  8 (22%) | 17 (46%)  10 (27%)  10 (27%) | p=0.880 | *26 (41%)*  *20 (32%)*  *17 (27%)* | *8 (80%)*  *1 (10%)*  *1 (10%)* | *p=0.074* | 4 (40%)  5 (50%)  1 (10%) | 17 (43%)  9 (23%)  14 (35%) | 13 (57%)  7 (30%)  3 (13%) | p=0.151 |
| APC  APC-low  APC-high | 56 (88%)  8 (13%) | 53 (79%)  14 (21%) | p=0.199 | *51 (91%)*  *5 (9%)* | *64 (79%)*  *17 (21%)* | *p=0.059* | 53 (86%)  9 (14%) | 60 (82%)  13 (18%) | p=0.606 | 98 (85%)  17 (15%) | 16 (76%)  5 (24%) | p=0.302 | 16 (89%)  2 (11%) | 59 (82%)  13 (18%) | 39 (85%)  7 (15%) | p=0.756 |
| NLR  NLR-low  NLR-high | 32 (54%)  27 (46%) | 32 (52%)  30 (48%) | p=0.773 | 29 (59%)  20 (41%) | 37 (48%)  40 (52%) | p=0.223 | **23 (42%)**  **32 (58%)** | **43 (62%)**  **26 (38%)** | **p=0.023** | 56 (53%)  49 (47%) | 11 (55%)  9 (45%) | p=0.891 | 10 (63%)  6 (37%) | 37 (54%)  31 (46%) | 20 (50%)  20 (50%) | p=0.695 |
| PLR  PLR-low  PLR-high | 32 (54%)  27 (46%) | 33 (54%)  28 (46%) | p=0.988 | 30 (61%)  19 (39%) | 39 (51%)  37 (49%) | p=0.277 | 26 (48%)  28 (52%) | 42 (61%)  27 (39%) | p=0.159 | 59 (57%)  45 (43%) | 10 (50%)  10 (50%) | p=0.579 | *13 (81%)*  *3 (19%)* | *36 (53%)*  *32 (47%)* | *20 (50%)*  *20 (50%)* | *p=0.083* |
| NPS  NPS0  NPS1  NPS2 | 28 (48%)  25 (43%)  6 (10%) | 27 (44%)  25 (41%)  9 (15%) | p=0.746 | 25 (51%)  20 (41%)  4 (8%) | 33 (43%)  32 (42%)  11 (15%) | p=0.504 | 24 (44%)  23 (43%)  7 (13%) | 34 (49%)  27 (39%)  8 (12%) | p=0.867 | 49 (47%)  43 (41%)  12 (12%) | 9 (45%)  8 (40%)  3 (15%) | p=0.910 | 10 (63%)  5 (31%)  1 (6%) | 29 (43%)  30 (44%)  9 (13%) | 19 (48%)  16 (40%)  5 (13%) | p=0.705 |

The relationship between clinicopathological features (including TME-based and SIR-related markers) and CMS immunohistochemistry (CK, FRMD6, CDX2, ZEB1, and HTR2b) was evaluated using Chi-squared test. For MMR evaluation nuclear expression was graded either positive or negative, similarly as with CDX2 and ZEB1. Cytoplasmic expression was categorized into low and high categories for CK and FRMD6, and into low, medium and high categories for HTR2B immunohistochemistry.

List of abbreviations: CK – cytokeratin, FRMD6 – framed 6, CDX2 – caudal type homeobox 2, ZEB1 – zinc finger E-box binding homeobox 1, HTR2B– 5-hydroxytryptamine receptor 2B, TSR – tumor-stroma ratio, KM grade – Klintrup-Makinen grade, GMS – Glasgow microenvironment score, CEA – carcinoembryonic antigen, CA 19-9 – carbohydrate antigen 19-9, CRP – C reactive protein, ANC – absolute neutrophil count, ALC – absolute lymphocyte count, APC -absolute platelet count, NLR – neutrophil-lymphocyte ratio, PLR – platelet-lymphocyte ratio, NPS – neutrophil platelet score, MMR – Mismatch repair
