# Supplementary material for: Microenvironment, systemic inflammatory response and tumor markers considering consensus molecular subtypes of colorectal cancer
Source: Pathol Oncol Res. 2024 Apr 5;30:1611574. doi: 10.3389/pore.2024.1611574 (PMC11026638; doi:10.3389/pore.2024.1611574)
Supplement: Supplementary file 6 [file DataSheet2.DOCX]

Supplementary table 2: List of primary antibodies and detection systems used during the immunohistochemical staining protocol.

| Function | Primary antibody (clone ID) | Manufacturer | Dilution | Incubation, primary antibody (minutes) | Post-primary antibodies | Incubation, post-primary (minutes) | Polymer | Incubation, polymer (minutes) | Incubation and DAB (minutes, vendor) | Counterstain |
| --- | --- | --- | --- | --- | --- | --- | --- | --- | --- | --- |
| Mismatch repair system (MMR) | MLH1  (G168-728) | CellMarque, Rocklin, CA, USA | 1:100 | 30 | BOND Polymer Refine Detection Kit, DS9800 (Leica, Weitzlar, Germany) | 15 | BOND Polymer Refine Detection Kit, DS9800 (Leica, Weitzlar, Germany) | 15 | 10,  Polymer Refine Detection Kit, DS9800 (Leica, Weitzlar, Germany) | Hematoxylin  4 minutes |
|  | MSH2 (DB15.82) | Diagnostic Biosystems, Pleasanton, CA, USA | 1:100 |  |  |  |  |  |  |  |
|  | MSH6  (EP49) | Eptomics, Burlingame, CA, USA | 1:75 |  |  |  |  |  |  |  |
|  | PMS2  (EP51) |  | 1:50 |  |  |  |  |  |  |  |
| Epithelial marker | CK (AE1/AE3) | Dako, Carpinteria, CA, USA | 1:150 |  |  |  |  |  |  |  |
| Instestinal marker | CDX2  (Dako-CDX2) |  | 1:50 |  |  |  |  |  |  |  |
| Hippo pathway activation | FRMD6  (PA5-9865) | Thermo Fisher Scientific, Waltham, MA, USA | 1:250 | 90 | Novolink Polymer DS, RE7150-CE (Leica, Weitzlar, Germany) | 30 | Novolink Polymer DS, RE7150-CE (Leica, Weitzlar, Germany) | 30 | 2-10,  Dako, Carpinteria, CA, USA |  |
| Epithelio-mesenchymal transition | ZEB1 (HPA027524) | Merck, Darmstadt, Germany | 1:500 |  |  |  |  |  |  |  |
| Serotonin receptor | HTR2B (HPA012867) |  | 1:250 |  |  |  |  |  |  |  |

For microsatellite instability evaluation the nuclear expression of MLH1, MSH2, MSH6 and PMS2 was graded either positive or negative, similarly as with CDX2 and ZEB1. Cytoplasmic expression was categorized into low and high categories for CK and FRMD6, and into low, medium and high categories for HTR2B immunohistochemistry.
