# Supplementary material for: Microenvironment, systemic inflammatory response and tumor markers considering consensus molecular subtypes of colorectal cancer
Source: Pathol Oncol Res. 2024 Apr 5;30:1611574. doi: 10.3389/pore.2024.1611574 (PMC11026638; doi:10.3389/pore.2024.1611574)
Supplement: Supplementary file 7 [file DataSheet8.DOCX]

Supplementary table 8: The relationship between stroma-tumor marker score and systemic inflammatory response

|  | Number of patients | STM 0  (mean rank) | STM 1  (mean rank) | STM 2  (mean rank) | p value |
| --- | --- | --- | --- | --- | --- |
| CEA | 135 | 58.97 | 75.56 | 101.13 | **<0.001** |
| CRP | 114 | 49.97 | 68.38 | 66.28 | **0.017** |
| Albumin | 82 | 45.98 | 36.20 | 34.82 | 0.115 |
| ANC | 127 | 60.15 | 69.93 | 67.27 | 0.370 |
| ALC | 127 | 65.94 | 62.54 | 56.55 | 0.697 |
| APC | 134 | 64.91 | 73.74 | 62.21 | 0.430 |

The relationship between stroma-tumor marker score (STM) and systemic inflammatory response related markers and CEA were evaluated using the non-parametric Kruskal-Wallis H test.

Significant variables were marked with bold font, tendencies where p<0.1 were marked with italic font.

Abbreviations: STM – stroma-tumor maker score, CEA – carcioembryonic antigen, CRP – C reactive protein, ANC – absolute neutrophil count, ALC – absolute lymphocyte count, APC, absolute platelet count.
