# Supplementary material for: Microenvironment, systemic inflammatory response and tumor markers considering consensus molecular subtypes of colorectal cancer
Source: Pathol Oncol Res. 2024 Apr 5;30:1611574. doi: 10.3389/pore.2024.1611574 (PMC11026638; doi:10.3389/pore.2024.1611574)
Supplement: Supplementary file 8 [file DataSheet1.DOCX]

Supplementary Table 1: Parameters of tumor characteristics and blood samples evaluated in the study

| Marker type | |  | Cutoff used for marker |
| --- | --- | --- | --- |
| TME | TSR, n=185 | TSR-low  TSR-high | less than 50% stromal content of hotspot |
|  | KM, n=185 | KM-low  KM-high | According to Klintrup et al. [1] |
|  | GMS, n=185 | GMS 0  GMS 1  GMS 2 | KM high  KM low and TSR low  KM low and TSR high |
| Consensus molecular subtypes,  n=155 | | CMS1: dMMR  CMS2/3: Epithelial  CMS4: Mesenchymal | According to Trinh et al. [2] |
| SIR | mGPS, n=95 | mGPS 0  mGPS 1  mGPS 2 | CRP-low, albumin-low  CRP-high, albumin-high  albumin-low and CRP-high |
|  | Albumin, n=107 | albumin-low  albumin-high | <35 g/l |
|  | CRP,  n=149 | CRP-low  CRP-high | <10 mg/l |
|  | ANC,  n=170 | ANC-low  ANC-high | <4.85 G/l |
|  | ALC,  n=170 | ALC-low  ALC-high | <1.65 G/l |
|  | APC,  n=180 | APC-low  APC-high | <400 G/l |
|  | NLR,  n=170 | NLR-low  NLR-high | <3 |
|  | PLR,  n=168 | PLR-low  PLR-high | <190 |
|  | NPS,  n=168 | NPS 0  NPS 1  NPS 2 | ANC-low, APC-low  ANC-high or APC-high  both high |
| Tumor markers | CEA,  n=155 | CEA low  CEA high | <5 ng/ml |
|  | CA19-9,  n=135 | CA19-9 low  CA19-9 high | <37 U/ml |
| Stroma-tumor marker score, n=135 |  | STM0  STM1  STM2 | TSR-low and CA19-9 low  TSR-low and CA19-9 high or  TSR-high and CA19-9 low  TSR-high and CA19-9 high |

Abbreviations: TME – tissue microenvironment, TSR – tumor-stroma ratio, KM grade – Klintrup-Makinen grade, GMS – Glasgow microenvironment score, CMS – consensus molecular subtype, dMMR – mismatch repair deficient, SIR – systemic inflammatory repsonse, mGPS – modified Glasgow prognostic score, CRP – C reactive protein, ANC – absolute neutrophil count, ALC – absolute lymphocyte count, NLR – neutrophil-lymphocyte ratio, PLR – platelet-lymphocyte ratio, NPS – neutrophil-platelet score, CEA – carcinoembryonic antigen, CA 19-9 – cancer antigen 19-9, STM – stroma-tumor marker score
